# Thyroidal expression of ER molecular chaperone GRP170 is required for efficient TSH-mediated thyroid hormone synthesis

**DOI:** 10.1172/jci.insight.191837

**Published:** 2025-09-09

**Authors:** Xiaohan Zhang, Crystal Young, Xiao-Hui Liao, Samuel Refetoff, Stephanie M. Mutchler, Jeffrey L. Brodsky, Teresa M. Buck, Peter Arvan

**Affiliations:** 1 Division of Metabolism, Endocrinology & Diabetes, and; 2 Department of Molecular & Integrative Physiology, University of Michigan, Ann Arbor, Michigan, USA.; 3 Department of Medicine and; 4 Pediatrics and Committee on Genetics, The University of Chicago, Chicago, Illinois, USA.; 5 Division of Renal Physiology, University of Pittsburgh School of Medicine, Pittsburgh, Pennsylvania, USA.; 6 Department of Biological Sciences, University of Pittsburgh, Pittsburgh, Pennsylvania, USA.

**Keywords:** Cell biology, Endocrinology, Protein traffic, Thyroid disease

## Abstract

Intracellular trafficking of secretory and membrane proteins from the endoplasmic reticulum (ER) to the cell surface, via the secretory pathway, is crucial to the differentiated function of epithelial tissues. In the thyroid gland, a prerequisite for such trafficking is proper protein folding in the ER, assisted by an array of ER molecular chaperones. One of the most abundant of these chaperones, Glucose-Regulated-Protein-170 (GRP170, encoded by *Hyou1*), is a noncanonical hsp70-like family member. Thyroid follicular epithelial cells abundantly express GRP170, but the role of this abundant ER chaperone in thyrocytes remains unknown. Here, we have examined the effect of inducible *Pax8*-specific (thyroid and kidney) deficiency of GRP170 in mice, in parallel with siRNA-treated PCCL3 (rat) thyrocytes for knockdown of GRP170. Thyrocyte-specific loss of GRP170 in vivo triggers primary hypothyroidism with a deficient thyroidal response to Thyroid-Stimulating Hormone (TSH). In addition, knockdown of GRP170 in PCCL3 thyrocytes inhibits the folding and forward trafficking of TSH receptors to the cell surface. Taken together, our findings suggest that GRP170 contributes to the conformational maturation of TSH receptors and thyroid gland responsiveness to TSH, which is required for proper regulation of thyroid hormone synthesis.

## Abstract 2

We show thyroidal loss of the endoplasmic reticulum chaperone GRP170 triggers primary hypothyroidism with thyrotropin resistance, attributable to defective intracellular trafficking of the thyrotropin receptor.

## Graphical abstract

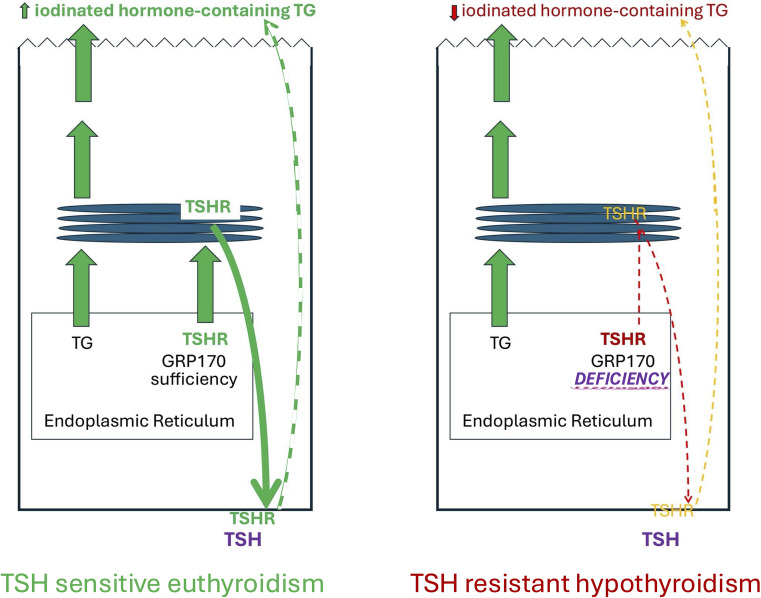

## Introduction

All epithelial tissues use the secretory pathway to deliver protein components selectively to the basolateral and apical plasma membrane surfaces, in addition to other post-Golgi destinations (1, 2). The thyroid gland combines these typical epithelial features with a commitment to produce immense quantities of a single secretory protein, thyroglobulin (TG) (3); its expression level is an order of magnitude higher than ubiquitously expressed housekeeping genes like actin (4). Nearly all TG is directed for apical release to the thyroid follicle lumen (5). Additionally, thyroid follicular epithelial cells (thyrocytes) synthesize the sodium-iodide symporter (NIS) and thyroid peroxidase (both under the influence of thyroid-stimulating hormone [TSH]) (6) for delivery to the basolateral and apical plasma membrane surfaces, respectively, as well as other thyroid-differentiated gene products (7, 8) that include the TSH receptor (TSHR) (9–12). All of these proteins are initially synthesized and folded in the endoplasmic reticulum (ER), where they are prepared for anterograde intracellular trafficking (13).

In general, only properly folded proteins are enabled for export from the ER (14, 15), and this process, which defines ER quality control (16), is assisted by ER molecular chaperones. The 2 best-studied classes of ER molecular chaperones are the hsp70 family member, BiP, and the lectin-like chaperones calnexin and calreticulin — both classes of chaperones interact with a very wide variety of protein substrates (although the lectin-like chaperones show obvious preference for N-linked glycoproteins) (17).

In contrast to BiP and the lectin-like chaperones, some other abundant ER chaperones such as GRP94 (14) and GRP170 (product of the *Hyou1* gene) (18, 19), despite being ubiquitously expressed, facilitate the biogenesis of a narrower selection of protein clients (20). GRP170 is of particular interest in the thyroid gland, as this tissue exhibits the second-highest expression of transcripts encoding GRP170 in the human body (testis/epididymis being first) according to both the Genome Tissue Expression portal (GTEx; https://www.gtexportal.org/home/) and HPA RNA-Seq datasets. Within the thyroid, immunolabeling indicates thyrocytes (https://www.proteinatlas.org/ENSG00000149428-HYOU1/tissue/thyroid+gland) as the cell type expressing the highest levels of GRP170. However, despite the reported preference of GRP170 for aggregation-prone substrates (presumably a feature of its unique extended substrate binding domain) (21), its key substrates in the thyroid are unknown. What is known at present is that GRP170 can be chemically cross-linked with WT TG (22, 23) as well as with misfolded mutant TG (24), yet it remains unknown whether GRP170 interaction with TG or another protein substrate is important for normal thyroid physiology.

It has recently been reported that, upon inducible *Pax8*-Cre–mediated deletion of *Hyou1*, pathological renal phenotypes develop, including a reduction in the levels of various cell surface ion channels and transporters in the proximal tubular epithelium (25). Although the kidney expresses high levels of *Pax8*, the highest expression of *Pax8* transcripts in the body are in thyrocytes. Thus, using the same inducible *Pax8*-Cre–mediated deletion, we have asked how GRP170 may influence the biogenesis of several of the major thyroid differentiated gene products — some of which include cell-surface membrane proteins, as well as the single predominant secretory protein, TG. Here we report, surprisingly, that TG folding and trafficking is not inhibited in GRP170-deficient thyroid tissue or thyrocytes in culture, yet animals with thyroid-specific loss of GRP170 develop TSH resistance (26), resulting in primary hypothyroidism.

## Results

### GRP170 is highly expressed in the thyrocyte ER.

**Figure 1:**
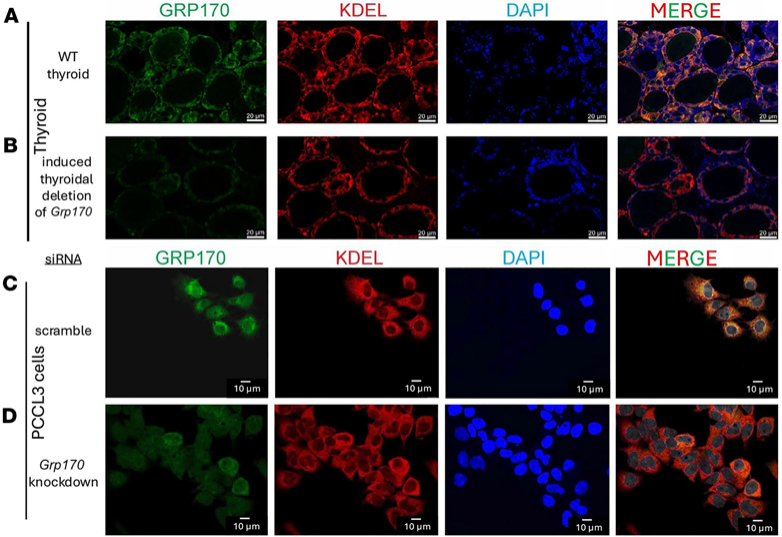
GRP170 is expressed in the ER of thyrocytes. (**A**) Immunofluorescence of control mouse thyroid costained for GRP170 (green), KDEL-containing proteins (red), DAPI (blue), and green/red merge (*n* = 5 animals examined). (**B**) Immunofluorescence of thyroid from mice with inducible *Grp170* deletion, immunostained as in **A** (*n* = 5 animals examined). (**C**) PCCL3 (rat thyrocyte) cells transfected (as a control) with scrambled oligo, examined by immunofluorescence for the same antigens, with presentation as those shown in **A**. (**D**) PCCL3 cells transfected with siRNA for knockdown of *Grp170*, examined by immunofluorescence as in **B**. Both **C** and **D** were performed in 3 independent transfection experiments. Scale bars: 20 μm (**A** and **B**), 10 μm (**C** and **D**).

GRP170 has been shown to exhibit hsp70-like chaperone activity as well as nucleotide exchange factor activity for BiP in yeast and model cell lines (19), yet the physiological importance of this abundant ER chaperone in tissues remains poorly understood (18). The GTEx portal indicates that bulk thyroid tissue in humans expresses 97.5 transcripts per million encoding GRP170. In tissue of (human and) mouse thyroid glands, GRP170 is distributed intracellularly in thyrocytes; we observed the protein to be concentrated in the cytoplasm with an immunolabeling pattern matching that of KDEL-containing proteins that serve as a marker of the ER lumen (Figure 1A). This labeling is almost completely lost after inducible gene deletion (Figure 1B), which is described further, below. An ER labeling pattern for GRP170 is also observed in PCCL3 cells (a rat thyrocyte line) in culture (Figure 1C). Thus, GRP170 is abundantly expressed in the ER of thyrocytes in vivo and in cell lines; after siRNA-mediated knockdown in PCCL3 cells, immunostaining of GRP170 is notably diminished (Figure 1D).

### Inducible Pax8-Cre–mediated deletion of GRP170 triggers hypothyroidism.

**Figure 2:**
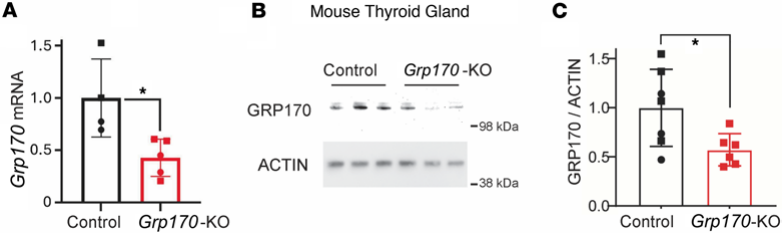
Genetic deletion of *GRP170* in mouse thyroid follicular epithelial cells. Mouse thyroid glands were harvested at 21 days after initiation of doxycycline treatment to induce *Pax8*-rtTA/LC1-Cre–mediated homozygous *Hyou1^fl/fl^* deletion between introns 1 and 24 (25). (**A**) Thyroidal *Grp170* mRNA levels measured by qPCR (normalized to *Tbp*; *n* = 4–5 animals per group; squares are males, circles are females; data are shown as mean ± SD, *t* test, *P < 0.05). (**B**) Immunoblotting of thyroid tissue lysates from the indicated genotypes with anti-GRP170 (actin is a loading control). (**C**) Quantitation of GRP170 protein levels from the indicated genotypes (*n* = 6–7 animals per group; squares are males, circles are females; data are shown as mean ± SD, *t* test, *P < 0.05).

**Figure 3:**
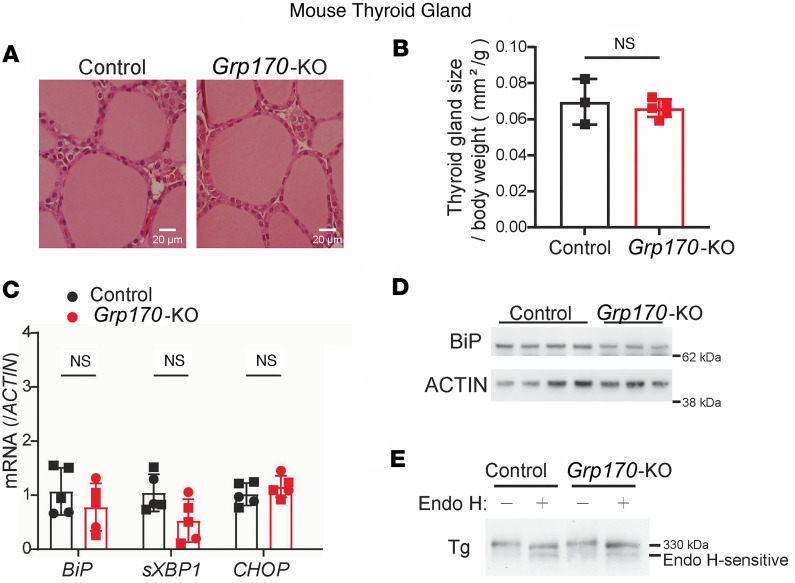
Thyroid tissue response to induced thyrocyte-specific loss of GRP170. (**A**) H&E staining of mouse thyroid tissue sections (*n* = 6–8 mice per group). Size bars: 20 μm. (**B**) Total thyroid gland size from the indicated genotypes (*n* = 3–5 per group; squares are males; data are shown as mean ± SD, *t* test). (**C**) Thyroidal *BiP,* spliced *Xbp1*, and *Chop* mRNA levels measured by qPCR from lysates of mice with thyroidal GRP170 deficiency, normalized to that of control animals (*n* = 5 per group; squares are males, circles are females; data are shown as mean ± SD, ANOVA). (**D**) Thyroidal BiP protein levels measured by immunoblotting in the indicated genotypes (*n* = 3–4 mice per group; actin is a loading control). (**E**) Immunoblotting of Tg protein from the indicated genotypes (*n* = 4 mice per group) after digestion with endoglycosidase H (+) or mock-digest (–). Endoglycosidase H sensitive and resistant bands are indicated.

To assess how GRP170 affects thyroid function, we used the previously described inducible *Pax8*-rtTA/LC-1 system for Cre-mediated LoxP-directed homozygous deletion between introns 1 and 24 in the *Hyou1* locus (25). To generate the inducible deletion, these animals were treated with doxycycline in the drinking water for 10 days. Bearing in mind that thyrocytes comprise ~50% of the cells in the mouse thyroid gland (27), after 21 days from the beginning of doxycycline treatment, thyroidal levels of mRNA encoding GRP170 dropped to ~30% of the control value (Figure 2A), and GRP170 protein (Figure 2B) dropped to ~50% (Figure 2, B and C), indicating efficient Cre-mediated gene deletion in thyrocytes. However, in contrast to the profound effects on kidney anatomy when GRP170 levels were similarly reduced (25, 28), thyroid histology was largely unaffected: follicles did not appear to be stimulated (Figure 3A), the size of the follicular lumen was not decreased (Supplemental Figure 1A; supplemental material available online with this article; https://doi.org/10.1172/jci.insight.191837DS1), actively dividing cells remained sparse (Supplemental Figure 1B), and there was no evidence of goiter development (Figure 3B).

Misfolding of thyroidal secretory proteins in the ER is associated with activation of the ER stress response (29–33). In both the kidney and mouse embryonic fibroblasts, markers of the ER stress response rose dramatically in response to GRP10 deficiency (25, 34). However, in the thyroid at 21 days after initiation of doxycycline treatment, we observed no increase of spliced *Xbp1* mRNA or of the mRNAs encoding BiP or CHOP (Figure 3C); moreover, thyroidal levels of BiP protein were not increased (Figure 3D), nor was there a change in the levels of phospho-eIF2α (Supplemental Figure 2). These data imply that deficiency of GRP170 is not associated with large-scale misfolding of proteins in the thyrocyte ER. This result was unexpected because TG, the major secretory protein of the thyroid gland, has been found to interact with GRP170 (22–24). We therefore examined TG trafficking in GRP170-deficient thyroid glands. Surprisingly, there was no increase in endoglycosidase H–sensitive (endo H–sensitive) (i.e., ER resident) TG in GRP170-deficient thyroid tissue (Figure 3E). Thus, when GRP170 is depleted, TG does not accumulate in the ER, unlike what has been observed in established models of TG misfolding (35–37).

**Figure 4:**
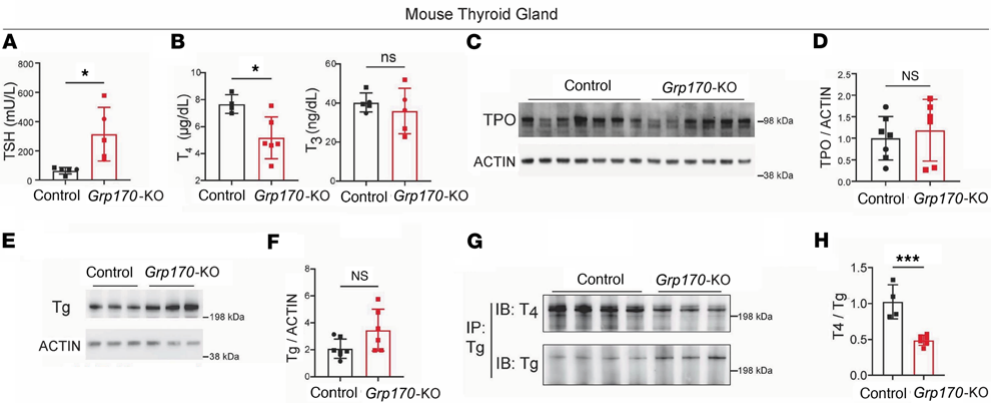
Primary hypothyroidism in mice with deficiency of GRP170 in thyrocyes. (**A**) Circulating TSH levels from control mice and those with induced deficiency of GRP170 at 21 days after initiation of doxycycline treatment (*n* = 5 animals per group; squares are males, circles are females). (**B**) Circulating levels of thyroxine (T_4_) and triiodothyronine (T_3_) from control mice and those with induced deficiency of GRP170 at 21 days after initiation of doxycycline treatment (*n* = 4–6 animals per group; squares are males, circles are females). (**C**) Immunoblotting of thyroid tissue lysates from the indicated genotypes with anti-TPO (actin is a loading control). (**D**) Quantitation of TPO protein levels from in lysates from mice with thyroidal GRP170 deficiency, normalized to that of control animals (*n* = 6–7 animals per group; squares are males, circles are females). (**E**) Immunoblotting of thyroidal TG protein levels from the indicated genotypes (these and the loading control actin come from the same samples as those shown in Figure 2B). (**F**) Quantitation of thyroidal TG protein levels from the indicated genotypes (*n* = 6–7 animals per group; squares are males, circles are females). (**G**) Immunoblotting of T_4_ content of thyroidal TG protein from the indicated genotypes (immunoblotting from the same samples with anti-TG in lower panel). (**H**) Quantitation of T_4_ content of TG protein in lysates from mice with thyroidal GRP170 deficiency, normalized to that of control animals (*n* = 4–6 animals per group; squares are males). Data are shown as mean ± SD, *t* test, *P < 0.05, ***P < 0.001.

In contrast to the lack of effects on TG and global ER homeostasis, circulating levels of TSH were significantly elevated after doxycycline-induced loss of GRP170 (Figure 4A), and circulating serum thyroxine (T_4_) levels were significantly decreased (Figure 4B). These findings link thyroidal GRP170 deficiency to primary hypothyroidism.

### Deficient thyroidal TSH response upon loss of GRP170.

Despite the increase of circulating TSH, there was no effect of the increase on the level of endogenous thyroid peroxidase in GRP170-deficient thyroid glands (TPO; Figure 4, C and D). We therefore sought to examine hormonogenesis within the TG protein, the precursor protein for the synthesis of T_4_ that derives exclusively from the thyroid gland (38). Remarkably, whereas TG was not diminished upon thyroid-inducible GRP170 deficiency (Figure 4, E and F), T_4_ formation within TG dramatically declined (Figure 4, G and H). Thus, the hypothyroidism induced by thyroidal deficiency of GRP170 is clearly caused by 1 or more steps leading to thyroid hormone formation.

**Figure 5:**
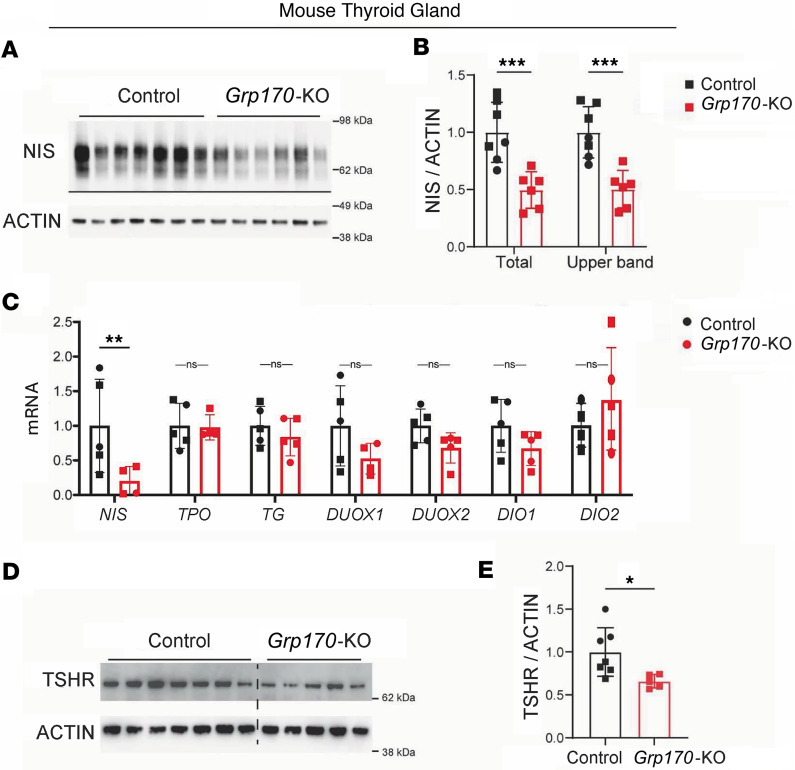
Deficiency of TSH receptors and NIS in the thyroid glands of mice with thyrocyte deficiency of GRP170. (**A**) Immunoblotting of NIS in thyroid tissue lysates from the inducible GRP170 deletion or controls (littermates lacking either *Pax8*-rtTA or LC-1). Animals of all genotypes were treated identically with doxycycline. Actin is a loading control. (**B**) Quantitation of either total NIS protein levels or only the upper NIS protein band in lysates from mice with thyroidal GRP170 deficiency, normalized to that of control animals (*n* = 6–7 animals per group; squares are males, circles are females; data are shown as mean ± SD, ANOVA, ***P < 0.001). (**C**) Thyroidal *Nis, Tpo,* *Tg*, *Duox1*, *Duox2*, *Dio1*, and *Dio2* mRNA levels (normalized to *Tbp*; *n* = 5 per group; squares are males, circles are females; data are shown as mean ± SD, ANOVA, *P < 0.05) were measured by qPCR from lysates of mice with thyroidal GRP170 deficiency, normalized to that of control animals. (**D**) Immunoblotting of thyroidal TSH receptor protein levels from animals like those described in **A**. Actin is a loading control. (**E**) Quantitation of thyroidal TSH receptor protein levels from animals in **D** (*n* = 5–7 animals per group; squares are males, circles are females; data are shown as mean ± SD, *t* test, *P < 0.05).

We noted that GRP170-deficient thyroid tissue exhibited a significant decrease of NIS protein levels (Figure 5, A and B). Additionally, in GRP170-deficient thyroid tissue, the electrophoretic mobility of the uppermost NIS protein band exhibited a subtle downward shift, which might conceivably reflect a small difference in Golgi-based modification of N-linked glycans (39–43). To better understand the basis for the decline in NIS levels, we examined *Nis* mRNA expression, which is tightly controlled by the action of thyrotropin on TSHRs (6, 44–46). Indeed, thyroidal *Nis* mRNA levels averaged only ~20% of control values (Figure 5C), despite the fact that circulating TSH was elevated ~5-fold above control levels (Figure 4A). Based on the response of WT C57BL/6J animals rendered hypothyroid by a 2-week treatment with propylthiouracil (PTU) (Supplemental Figure 3A), we would expect upregulated expression of genes like *Tpo* and *Nis* (Supplemental Figure 3B). However, none of the genes that might be expected to positively respond to TSH stimulation were upregulated in GRP170-deficient thyroid glands (Figure 5C). Moreover, by immunoblotting, thyroidal TSHR protein levels appeared diminished in GRP170-deficient thyroid glands (Figure 5, D and E). Together, these data suggest the hypothesis that thyroidal GRP170 deficiency adversely affects TSHRs, leading to thyrotropin resistance (26, 46).

### Effect of GRP170 deficiency in a cultured rat thyroid epithelial cell line.

**Figure 6:**
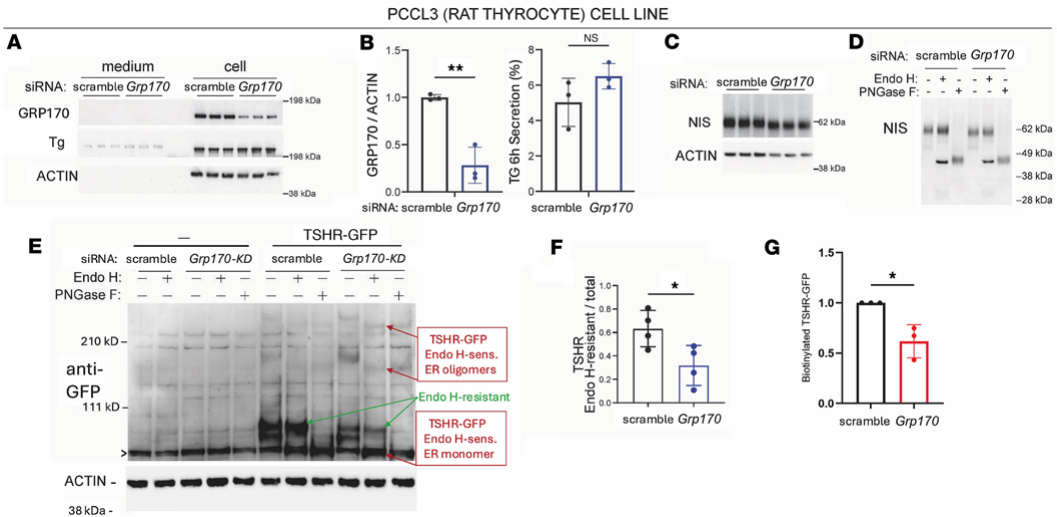
Phenotype of thyrocyte-specific deficiency of GRP170 in PCCL3 cells. (**A**) Immunoblotting of GRP170 and TG content of cells (last 6 lanes) and medium collected for 6 hours (first 6 lanes) at 48 hours after siRNA-mediated knockdown of GRP170, or scrambled oligo control. Actin is a loading control. (**B**) Quantitation of GRP170 protein levels and TG secretion from the indicated samples (*n* = 3 independent transfections in each group; data are shown as mean ± SD, *t* test, **P < 0.01). (**C**) Immunoblotting of NIS protein at 48 hours after siRNA-mediated knockdown of GRP170 (*n* = 3 transfections per group). Actin is a loading control. (**D**) Digestion of NIS protein with endoglycosidase H, or PNGase F in the samples as indicated (*n* = 4 transfections per group). (**E**) PCCL3 cells were transfected either with empty vector (–) or to express TSHR-GFP as indicated; after 24 hours, the cells were transfected with siRNA for knockdown of GRP170 or scrambled oligo control. At 48 hours after knockdown, the cells were lysed, digested with endoglycosidase H or PNGase F or were mock-digested, analyzed by 3%–8% Tris-Acetate NuPAGE, electrotransferred to nitrocellulose, and immunoblotted with anti-GFP antibodies. Endo H–sensitive forms are indicated with up-sloped red arrows; endo H–resistant forms are indicated with down-sloped green arrows. (**F**) Quantitation of the fraction of endo H–resistant TSHR-GFP from the indicated samples (*n* = 4 independent transfections in each group; data are shown as mean ± SD, *t* test, *P < 0.05). (**G**) Quantitation of surface-biotinylated TSHR-GFP in PCCL3 cells from the experiments shown in Supplemental Figure 4 (data are shown as mean ± SD, *t* test, *P < 0.05).

To test this hypothesis and more directly examine the effect of GRP170 deficiency on intracellular protein trafficking in thyrocytes, we employed PCCL3 cells, a rat thyrocyte cell line. Three days after siRNA-mediated knockdown to induce GRP170 protein deficiency (Figure 1, C and D, and Figure 6, A and B), neither intracellular TG protein levels nor TG secretion into the culture medium were impaired (Figure 6A). Under these conditions, total NIS protein levels were not altered but NIS protein exhibited a downward shift in its electrophoretic mobility (Figure 6C), which is consistent with a defect in the TSH-dependent effect on Golgi-based modification of N-linked glycans (39–43). Consistent with this interpretation, in both the endo H–sensitive form of NIS, and after PNGase F digestion to remove all N-linked glycans, the difference in NIS electrophoretic mobility in GRP170-deficient thyrocytes was abolished (Figure 6D).

Because the commercial anti-TSHR antibody did not cross-react in rat thyrocytes, we expressed TSHR-GFP upon plasmid transfection, as commonly reported by others (9–12). After 24 hours, siRNA or scrambled oligos were transfected, and the effect of GRP170 deficiency was examined after a further 48 hours. Although only ~10% of PCCL3 thyrocytes were transfected with the TSHR-GFP plasmid, by immunoblot analysis with anti-GFP after Tris-acetate NuPAGE, control PCCL3 cells not expressing TSHR-GFP revealed only background bands (including a major nonspecific band marked with a caret; Figure 6E). However, thyrocytes expressing GFP-tagged TSHRs revealed at least 4 distinct specific bands.

When GRP170 was present, the most predominant of the TSHR-GFP bands corresponded to a monomer (~100 kDa) that was resistant to digestion with endoglycosidase H; these represent molecules that underwent successful anterograde trafficking from the ER to acquire Golgi/post-Golgi glycan modifications (Figure 6E). The next most abundant form was a slightly faster-migrating form that was endoglycosidase-sensitive; unfortunately, after endo H digestion, this sensitive band migrated at the same position as the nonspecific band (thereby enlarging this band; Figure 6E). In GRP170-deficient thyrocytes, this TSHR-GFP form (that had not advanced to acquire Golgi carbohydrate modifications) was the predominant band (Figure 6E). Moreover, there were at least 2 higher molecular weight forms of TSHR-GFP, and these were comprised of entirely of endo H–sensitive molecules (Figure 6E), suggesting misfolded oligomers incompetent for export from the ER. The net effect was that GFP-tagged TSHRs were less efficiently exported from the ER when GRP170 was deficient compared with control cells with normal levels of GRP170 (Figure 6F). For more direct evidence of diminished plasmalemmal TSHR-GFP, we biotinylated PCCL3 cell-surface proteins with nonpermeant sulfo-NHS-biotin and, after cell lysis, precipitated the surface-labeled proteins with streptavidin-agarose. Western blot analyses revealed cell surface TSHR-GFP that was identical to the endo H–resistant band, and this was diminished in GRP170-deficient PCCL3 cells (Figure 6G and Supplemental Figure 4). Altogether, the data suggest that the presence of GRP170 improves TSHR folding efficiency, which leads to its trafficking from the ER through the secretory pathway to the cell surface.

**Figure 7:**
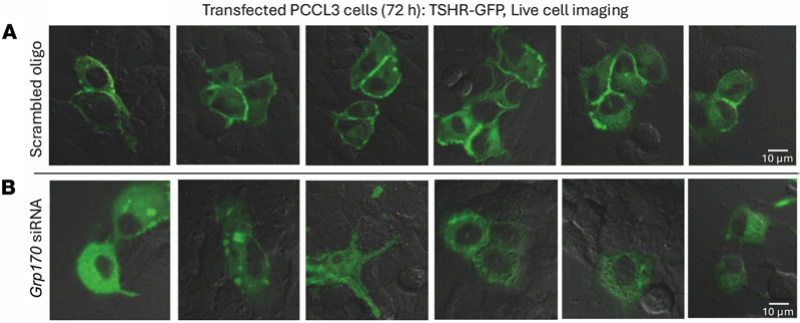
Trafficking of GFP-tagged TSH receptors by live cell imaging in PCCL3 cells. PCCL3 cells were transfected to express TSHR-GFP and, 24 hours later, transfected with siRNA for knockdown of GRP170 or scrambled oligo control exactly as in Figure 6E. (**A**) At 48 hours after the second transfection, TSHR-GFP distribution was imaged in live control PCCL3 cells. (**B**) At 48 hours after the second transfection, TSHR-GFP distribution was imaged in live PCCL3 cells deficient for GRP170. The results in **A** and **B** were repeated and confirmed (3 independent transfection experiments).

To further support this conclusion, we used live-cell imaging of TSHR-GFP–transfected PCCL3 thyrocytes. In cells with normal levels of GRP170 (transfected with only scrambled oligos), cell-surface TSHR-GFP fluorescence was apparent in addition to cytoplasmic fluorescence (Figure 7A). In GRP170-deficient PCCL3 cells, TSHR-GFP fluorescence at the cell surface was diminished; instead, fluorescence was distributed throughout the cytoplasm (as would be expected for an ER distribution), and some of the cells contained fluorescent cytoplasmic puncta, which might represent accumulation of misfolded forms (Figure 7B). These findings in GRP170 replete and depleted cells were independent of the strength of the GFP fluorescence signal, suggesting that these behaviors are not tightly linked to TSHR-GFP expression level. In conclusion, our data indicate that GRP170 deficiency in thyrocytes leads to a diminished presence of TSHR at the cell surface, thereby accounting for primary hypothyroidism associated with thyroidal TSH resistance.

## Discussion

We have sought to better understand the synthesis, folding, and trafficking of TG, the abundant precursor protein that has been evolutionarily selected for efficient thyroid hormone synthesis over the past 500 million years (47). TG is the overwhelmingly predominant thyroid translation product, and its large size and globular structure renders it exquisitely sensitive to misfolding that can be initiated in any of its peptide regions (each region generally composed of repeating domains) (38, 48). This can account for the large array of pathogenic mutations — including hundreds of missense mutants throughout the molecule — that result in hypothyroidism caused by TG misfolding and entrapment within the ER (49).

GRP170 is highly expressed in thyrocytes (Figure 1), and recent proteomic studies with stringent washing conditions have reported that GRP170 can be cross-linked to TG in the ER (23). Using similar methodology, the TG-A2234D and TG-C1264R mutants have been reported to exhibit even greater and more prolonged interaction with GRP170 (24). These reports suggest the possibility that this abundant ER chaperone may recognize 1 or more incompletely folded forms of TG during its conformational maturation (such folding is essential to normal thyroid physiology, by enabling TG to pass ER quality control for intracellular trafficking, which in turn leads to TG secretion and hormonogenesis within the follicle lumen) (50, 51). In the present study, our data establish that tissue-specific genetic deletion leading to loss of GRP170 (Figure 2) triggers primary hypothyroidism with decreased circulating T_4_ levels and a compensatory increase in circulating TSH (Figure 4, A and B). However, several independent lines of evidence indicate that GRP170 is not critical for preparing newly synthesized TG for its physiological role in thyroid hormone synthesis.

First, upon GRP170 depletion in the thyroid gland, we did not detect any decrease of total TG or endo H–resistant TG or shrinkage of the follicle lumen (Figure 3E; Figure 4, E and F; and Supplemental Figure 1A) — features that invariably accompany TG misfolding (38). Second, TG misfolding triggers activation of the ER stress response with several well-defined upregulated markers; however, thyroidal GRP170 deficiency does not reveal an increase in these markers (Figure 3, C and D, and Supplemental Figure 2). Third, TG misfolding is typically accompanied by ER swelling that leads to cell swelling, which is seen even in heterozygous animals bearing only 1 mutant *TG* allele along with one WT (33); nevertheless upon loss of GRP170, thyroidal histology is unchanged (Figure 3A) and the thyrocyte cytoplasm does not enlarge (Figure 1, B and C). Indeed, upon inducible GRP170 deficiency, the endo H–resistant pool of TG, which is contained almost entirely in the follicle lumen, is undiminished, and its mobility by SDS-PAGE is identical to that in control thyroid tissue (Figure 3E). Moreover, after siRNA-mediated GRP170 knockdown in PCCL3 cells, TG secretion is unimpaired (Figure 6A). Thus, although GRP170 and TG can interact in the ER (23), anterograde trafficking of TG (which is dependent upon successful TG folding) does not appear to be dependent upon its interaction with GRP170 either in vivo or in thyrocyte cell culture. Nevertheless, TG folding almost certainly requires interactions with ER chaperones and oxidoreductases (50, 52, 53), and there may be compensatory redundancy in these systems (54).

Although TG appears unaffected, animals with inducible loss of thyroidal GRP170 develop primary hypothyroidism (Figure 4, A and B), which can already be detected by a diminished T_4_ content in thyroidal TG (Figure 4, G and H). Our collective evidence strongly suggests that the thyroid defect originates from thyroidal resistance to TSH, which is known to cause a decrease in iodinated TG (46). First, TSHR protein expression appears diminished in inducible GRP170-deficient thyroid glands (Figure 5, D and E). Second, despite a 5-fold elevation of TSH (Figure 4A), there was no evidence of thyroid gland growth after GRP170 deletion (Figure 3B and Supplemental Figure 1B) and no increased expression of any TSH-dependent thyroid genes (Figure 5C) that are known to be upregulated in primary hypothyroidism (Supplemental Figure 3). Indeed, there was a marked decrease of *Nis* mRNA and protein expression (Figure 5, A–C). We note that, of all thyroid differentiated genes, *Nis* is the most highly dependent upon TSH signaling (6, 45, 46). Additionally, there was no increase of TPO protein expression (Figure 4, C and D). Finally, there was a subtle electrophoretic mobility shift of NIS protein in GRP170-deficient thyrocytes in vivo (Figure 5A) and in vitro (Figure 6C), compatible with a change in Golgi N-glycan modifications that are known to be under the influence of TSH (39–43).

Animals with inducible Pax8-Cre–mediated Grp170-KO do develop renal insufficiency, and it is reasonable to entertain the hypothesis that these animals might experience the “euthyroid-sick” syndrome rather than primary hypothyroidism. However, euthyroid-sick syndrome (55) (a) typically exhibits circulating TSH that is normal or low, rather than elevated as seen here; (b) has not been shown to be associated with diminished iodination of (and thyroid hormone formation within) TG, although this does occur in states of TSH resistance (46), which was observed in the present study; and (c) is not associated with diminished thyroidal response to TSH. Additionally, our findings in PCCL3 cells directly demonstrate folding and trafficking problems (and cell surface expression levels) of recombinant TSHRs in cells with knockdown of *Grp170* — which parallel the in vivo findings yet are wholly unrelated to any issues of renal function.

Indeed, the simplest explanation to account for all of our data is that GRP170 promotes the folding and trafficking efficiency of the TSHR. Expression of GFP-tagged TSHRs in PCCL3 cells ordinarily leads to a majority of the receptors being successfully exported from the ER and acquiring endo H–resistance (Figure 6, E and F) to reach the cell surface (Figure 6G and Figure 7A). However, upon siRNA-mediated knockdown to reduce GRP170 protein expression, only a minority of the expressed TSHR acquires endo H–resistance (Figure 6F); the majority is recovered as either an endo H–sensitive monomeric species or SDS-resistant higher molecular weight oligomers that are also entirely endo H–sensitive (Figure 6E).

The efficiency of trafficking of TSHRs via the secretory pathway to the cell surface is directly related to TSHR function (56). Although immunofluorescence analyses do not precisely quantify the fraction of all recombinant TSHR molecules that reside at the plasma membrane (in part due to the limits of resolution of immunofluorescence microscopy) (57), such a decrease at the cell surface is visually apparent in thyrocytes deficient for GRP170 (Figure 7B) and is also reflected by decreased surface biotinylation of TSHRs in thyrocytes (Figure 6G and Supplemental Figure 4). Diminished surface expression of TSHRs is linked to TSH resistance (12). Moreover, entrapment of incompletely folded TSHRs in the ER is likely to result in their enhanced degradation, which may explain why, when GRP170 was depleted, thyroidal TSHR protein levels appeared at only ~50% of control values (Figure 5, D and E).

We note that a large number of TSHR loss-of-function mutants have been reported to trigger primary hypothyroidism with TSH resistance in humans, including many missense mutants (26). While some mutants may be deficient in TSH signal transduction, many such mutants exhibit impaired overall receptor expression level (26). There is a strong possibility that some (if not all) of these “poorly expressed” TSHR mutants may exhibit defective folding and anterograde trafficking in the secretory pathway. In our current study, the data highlight that, rather than direct mutations in thyroid differentiation genes themselves, defects in genes involved in maintaining ER homeostasis in thyrocytes can also adversely affect the folding and trafficking of key proteins involved in thyroid hormonogenesis — such as the TSHR — and, in that way, confer genetic risk of hypothyroidism.

## Methods

### Sex as a biological variable.

Our findings are expected to be relevant for both sexes; therefore, data from both males and females were included, with sex of each animal indicated in graphs.

### Mice.

All mice were in a C57BL/6J background.

Mice were maintained under standard conditions. *Hyou1^fl/fl^/Pax8-rtTA*/LC-1 mice and controls (littermates lacking either *Pax8*-rtTA or LC-1) were generated in the C57BL/6J background as previously described (25). Genotyping was performed by Transnetyx with the following PCR primers:

HYOU1 (*Grp170*) floxed allele (5′–3′): forward (F): CGTATAATGTATGCTATACGAAGTTATGGCTTGTAACC, reverse (R): CTGGCACAGAAGGGTTTGTCC; WT HYOU1 (*Grp170*) allele: F: GGATCTTCCACCTTCGTCAGGT, R: GCTGCCTACGCAGACGTTGT; CRE (LC-1): F:TTAATCCATATTGGCAGAACGAAAACG, R: CAGGCTAAGTGCCTTCTCTACA; and Pax8-rtTA: F: ACAGTACGAAACCCTGGAAAATCAG, R: GCGGACAGAGCGTACAGT.

To induce Cre-mediated gene deletion, mice were administered Doxycycline (2 g/L) in water containing 5% sucrose for 10 days as previously described (25). After euthanasia, thyroid glands were removed and either frozen in liquid nitrogen or fixed in formalin. For PTU treatment, WT mice were fed low-iodide chow containing PTU (1.5g/kg, Envigo TD.95125) for 2 weeks.

### Primary antibodies.

Primary antibodies included rabbit anti-TG (365997, Santa Cruz Biotechnology Inc.); mAb anti-TG (ab156008, Abcam); rabbit anti-GRP170, rabbit anti-TPO, and rabbit anti-BiP (previously described in refs. 25, 35, 58–60); mouse anti-KDEL (ADI-SPA-827-D, Enzo); anti-T_4_ (1H1) (sc-52247, Santa Cruz Biotechnology Inc.); anti-TSHR (14450-1-AP, Proteintech); anti-GFP (RGFP-45A-Z, Immunology Consultants Laboratory); and mouse anti-Actin (66009-1-Ig, Proteintech). PA716 polyclonal rabbit anti-rat NIS (rNIS) from the lab of B. Rousset (Faculté de Médecine Lyon-Est., Lyon, France) was shared by S. Jhiang and M. Ringel (Ohio State University, Columbus, Ohio, USA).

### Serum measurements.

Serum total TSH and T_3_ concentrations were measured using radioimmunoassays as previous described (61, 62). Total T_4_ was assayed by ELISA (Diagnostic Automation/Cortez Diagnostics).

### Thyroid gland size measurement.

Thyroid gland area (mm^2^, normalized to body weight in grams) was measured and quantified as previously described (31). Measurement of follicle luminal size was performed using AIVIA Artificial Intelligence-guided Software.

### PCR.

Total RNA from the mouse thyroids was isolated using RNeasy Plus Kit (Qiagen) followed by cDNA synthesis using High-Capacity cDNA Reverse Transcription Kit (Applied Biosystems). For real-time PCR, either Radiant Green Hi-ROX (Alkali Scientific) or PowerUp SYBR-Green master mix (Applied Biosystems) was used for quantitative PCR (qPCR) on a StepOnePlus thermocycler (Thermo Fisher Scientific). Gene expression was normalized to *Tbp* mRNA, with primers as follows: *Grp170/Hyou1* (F: 5′-CCTGAGGATCTTCGGGTATTTG-3′; R: 5′-CTTTGGACTCATAGTCGGGATATT-3′), *Bip/Hspa5* (F: 5′-GGATCATCAATGAGCCTACAGC-3′; R: 5′-ACCCAGGTCAAACACAAGGAT-3′), spliced *Xbp1* (F: 5′-GGTCTGCTGAGTCCGCAGCAGG-3′; R: 5′-GAAAGGGAGGCTGGTAAGGAAC-3′), *Chop/Ddit3* (F: 5′-CCTGAGGAGAGAGTGTTCCAG-3′; R: 5′-GACACCGTCTCCAAGGTGAA-3′), *Atf4* (F: 5′-GCAAGGAGGATGCCTTTTC-3′; R: 5′-GTTTCCAGGTCATCCATTCG-3′), *Nis* (F: 5′-GGGACGCTGCAGTACTTGGT-3′; R: 5′-CAGACCACGGCCTTCATACC-3′), *Tpo* (F: 5′-GAGTCAGGGGAGGGGGATTT-3′; R: 5′-GGCAGCTTGAGACTTGCCTT-3′), *Tg* (F: 5′-TGATCTGATCCACAACTACAACAG-3′; R: 5′-ATTCCAGTCCTGTCTCAGCC-3′), *Duox1* (F: 5′-GAGCTTCCAGGAGTGGGAAC-3′; R: 5′-ACGCACTTTGGTCGAGGAAT-3′), *Duox2* (F: 5′-GTCTCCAATGTGGACCCCAG-3′; R: 5′-ATCACAGTAACAGGCGCACA-3′), *Dio1* (F: 5′-GTTTGTCCTGAAGGTCCGCT-3′; R: 5′-AGGTGCAACTGCCAAAGTTC-3′), *Dio2* (F: 5′-CTTCTGAGCCGCTCCAAGTC-3′; R: 5′-GGGAGCATCTTCACCCAGTT-3′); *Tbp* (F: 5′-CCTTGTACCCTTCACCAATGAC-3′; R: 5′-ACAGCCAAGATTCACGGTAGA-3′); GFP (F: 5′-ATCTTCTTCAAGGACGAC-3′; R: 5′- TTGTGGCTGTTGTAGTTG-3′).

### Cell culture and transfection.

PCCL3 cells (from B. DiJeso, University of Salento) were cultured in DMEM/F-12 with 5% bovine calf serum and a 4-hormone (MilliporeSigma) mixture containing 1 mIU/mL TSH, 1 μg/mL insulin, 1 nM hydrocortisone, and 5 μg/mL apo-transferrin. PCCL3 cells were transiently transfected using ViaFect (Promega) to express the human TSHR tagged at the C-terminus with GFP, yielding an active full-length receptor migrating between 85 and 110 kDa (9, 10). Cells were analyzed at 72 hours after transfection.

For siRNA-mediated knockdown of GRP170 in a rat PCCL3 cell line, transfection was performed at 30% confluency using Lipofectamine RNAiMAX (Invitrogen) with the following oligo (20nM): 5′-GAGAUGGACCAGAUCUUCAtt-3′ (Silencer Select; Thermo Fisher Scientific). In some experiments, cells were retransfected after 24 hours. Cells were collected and analyzed at 48 hours for single-transfection and 72 hours for double-transfection.

For live cell imaging, PCCL3 cells were cultured in LabTek-II coverglass chambers (Nunc). At 72 hours after transfection (48 hours after knockdown of GRP170), green fluorescence of TSHR-GFP was examined using a Nikon A1 Confocal Microscope.

### Biotinylation of cell surface proteins and detection of cell surface TSHR-GFP in PCCL3 cells with knockdown of GRP170.

PCCL3 cells (30% confluent) were transfected to express TSHR-GFP and, 24 hours later, transfected with scrambled oligo or siRNA for knockdown of GRP170 as describe above. At 48 hours after siRNA transfection, confluent monolayers of PCCL3 cells were washed with ice-cold PBS 3 times and labeled with EZ-Link Sulfo-NHS-SS-Biotin (Thermo Fisher Scientific) at 0.6 mg/mL in label buffer (0.25M sucrose, 2 mM CaCl_2_, 0.5 mM MgCl_2_, 10 mM triethanolamine, pH 8.5) for 30 minutes on ice. Biotinylation was quenched with ice-cold Tris (25 mM, pH 8.0) for 8 minutes on ice, followed with washing twice with ice-cold PBS and lysis in IP buffer (25 mM Tris·HCl [pH 7.4], 150 mM NaCl, 1% NP-40, 1 mM EDTA, 5% glycerol with protease/phosphatase inhibitor; Thermo Fisher Scientific). Biotinylated proteins were pulled down with Pierce streptavidin-agarose (Thermo Fisher Scientific), incubated overnight at 4°C. Precipitates were washed 3 times in IP buffer, incubated at 37°C for 30 minutes in SDS-gel sample buffer (Invitrogen) containing 50 mM DTT, resolved electrophoretically by 4%–12% acrylamide gradient NuPAGE, transferred to nitrocellulose, and immunoblotted with rabbit anti-GFP.

### Preparation and immunostaining of thyroid sections.

Mouse thyroids were quickly dissected and immersion-fixed in 10% formalin, paraffin embedded, sectioned, and stained with H&E (Vector). For immunofluorescence, thyroid sections were deparaffinized with Citrisolv (Thermo Fisher Scientific) and a series of ethanol, followed by antigen retrieval in citrate buffer and blocking with 1.5% goat serum. For PCCL3 cells, cells cultured in Lab-Tek II Chamber Slides were fixed with 10% formalin, permeabilized with 0.4% Triton X-100 for 20 minutes, and blocked with 3% BSA for 1 hour. Thyroid sections and PCCL3 cells were incubated in primary antibody at 4°C overnight and Alexa Fluor–conjugated secondary antibodies (Invitrogen, A21422, A11008) at room temperature for 1 hour, followed by counterstaining with ProLong Gold and DAPI (Invitrogen) and imaging with Nikon A1 confocal microscope or Leica STELLARIS 8 FALCON confocal microscope. For IHC of Ki67, VECTASTAIN-ABC (Vector Laboratories) was used and imaged with Olympus EX51 Microscope.

### Western blotting and immunoprecipitation.

Mouse thyroid glands or PCCL3 cells were lysed in RIPA buffer (150 mM NaCl, 25 mM Tris-HCl [pH 7.6], 1% NP-40, 1% sodium deoxycholate, 0.1% SDS; Thermo Fisher Scientific) supplemented with protease and phosphatase inhibitor cocktails (Thermo Fisher Scientific). Protein concentrations were determined by the Pierce BCA protein assay kit (Thermo Fisher Scientific). Protein lysates were either heated to 95°C for 5 minutes or, when analyzing NIS and TPO, to prevent aggregation, samples were warmed to 37°C for 30 minutes in SDS-gel sample buffer (Invitrogen) with 50 mM DTT. Samples were then resolved by SDS-PAGE, transferred to nitrocellulose membranes, blocked with 5% milk, and immunoblotted with the indicated primary antibodies and appropriate HRP-conjugated secondary antibodies (Bio-Rad, 1721019,1706516). Bands were visualized by enhanced chemiluminescence and quantified using ImageJ (NIH). For immunoprecipitation, thyroid glands were homogenized in IP lysis buffer (25 mM Tris·HCl [pH 7.4], 150 mM NaCl, 1% NP-40, 1 mM EDTA, 5% glycerol) plus protease inhibitor cocktail (Roche). Thyroid homogenates were incubated with rabbit polyclonal anti-TG antibody and protein A-agarose (Invitrogen) overnight at 4°C. Precipitates were washed 3 times in IP lysis buffer, boiled in SDS gel-sample buffer containing 50 mM DTT, resolved by SDS-PAGE, transferred to nitrocellulose membrane, and immunoblotted with mouse anti-T_4_ and rabbit mAb anti-TG.

### Endoglycosidase-H and PNGase-F digestion.

Thyroid homogenates or PCCL3 lysates were treated in denaturing buffer (New England Biolabs) at 37°C for 30 minutes and then digested with either endoglycosidase H (1,000 units, New England Biolabs), PNGase F (500 units, New England Biolabs), or mock digested at 37°C for 1 hour.

### Statistics.

Comparisons between 2 groups were made by unpaired 2-tailed Student’s *t* test. Multiple comparisons were made by 2-way ANOVA with Šídák’s correction. All statistical analyses were conducted with GraphPad Prism. Data are represented as mean ± SD; *P* < 0.05 was considered significant.

### Study approval.

All animal experiments performed were in compliance with and approved by the IACUC at the University of Pittsburgh (IACUC protocol no. 21038908).

### Data availability.

Values for all data points in graphs are reported in the Supporting Data Values file. Materials are freely available upon request.

## Author contributions

XZ and PA designed experiments; XZ and CY performed experiments; TMB, SMM, and XHL provided assistance; TMB, JLB, and SR provided key reagents; XZ wrote methods; PA supervised the work and wrote the manuscript; and all coauthors reviewed, edited, and approved the manuscript.

## Supplementary Material

Supplemental data

Unedited blot and gel images

Supporting data values

## Version 1 09/09/2025

Electronic publication
