# Supplementary material for: Thyroidal expression of ER molecular chaperone GRP170 is required for efficient TSH-mediated thyroid hormone synthesis
Source: JCI Insight. 2025 Sep 9;10(17):e191837. doi: 10.1172/jci.insight.191837 (PMC12487683; doi:10.1172/jci.insight.191837)
Supplement: Supplemental data [file jciinsight-10-191837-s108.pdf]

Supplemental Figure Legends

Supplemental Fig. S1. **Thyroid follicles in mice with genetic deletion of *GRP170* in thyroid follicular epithelial cells.** A) Mouse thyroid gland sections were stained with hematoxylin and eosin; all sections were imaged at the same magnification, and the luminal area of each thyroid follicle quantified by Imaris software. Each color is a different mouse (n=6-8 mice per group), each point is a different thyroid follicle. Mean values are indicated (t-test, ns = non-significant). B) Mouse thyroid gland sections were stained for Ki67 with immunoperoxidase from the indicated genotypes (n=6-8 mice per group). In most sections, no Ki67-positive cells were detected; in each of the two sections shown in the Figure, two dividing cells are seen per field. A scale bar is indicated.

Supplemental Fig. S2. **Immunoblotting of thyroid tissue in mice with genetic deletion of *GRP170* in thyroid follicular epithelial cells.** Immunoblotting for GRP170 (*upper panel*), KDEL proteins (*middle panel*), and phospho-eIF2α (lower panel) are shown (n=3 independent transfections in each group). This analysis and the loading control (actin) are derived from the same samples as those shown in Fig. 6A.

Supplemental Fig. S3. **Transcriptional response of TPO and NIS to primary hypothyroidism.** C57BL6/j mice (4 animals per group; squares=males, circles=females) were fed normal chow (black bars) or low-iodide chow containing PTU (red bars) for two weeks to generate hypothyroidism with low circulating T_4 (panel A; n=5-7 per group, t-test, ****p<0.0001). Thyroidal RNA was reverse transcribed and qRT-PCR was performed with specific primer pairs; the TPO and NIS mRNA levels in +PTU animals were normalized to the normal chow control animals (panel B, n=4 per group, ANOVA, **p<0.01****p<0.0001).

Supplemental Fig. S4. **Surface expression of TSH receptor in PCCL3 thyrocytes with deficiency of GRP170.** In three independent experiments, PCCL3 cells were either untransfected or plasmid-transfected to express TSHR-GFP. Thereafter, the cells were re-transfected with scrambled oligo or siRNA for knockdown of GRP170 (see Methods). In five independent preliminary transfections comparing scrambled oligo to siRNA for knockdown of GRP170, we determined that the expression level of TSHR-GFP mRNA was undiminished in cells with knockdown of GRP170 (and no TSHR-GFP mRNA could be detected in cells untransfected with plasmid DNA). As shown in the Figure in three further independent experiments, PCCL3 cells were surface-biotinylated before lysis, and the surface-biotinylated proteins precipitated with streptavidin agarose. Streptavidin precipitates were resolved by SDS-PAGE and Western blotting with anti-GFP; biotinylated TSHR-GFP is indicated (red arrow).

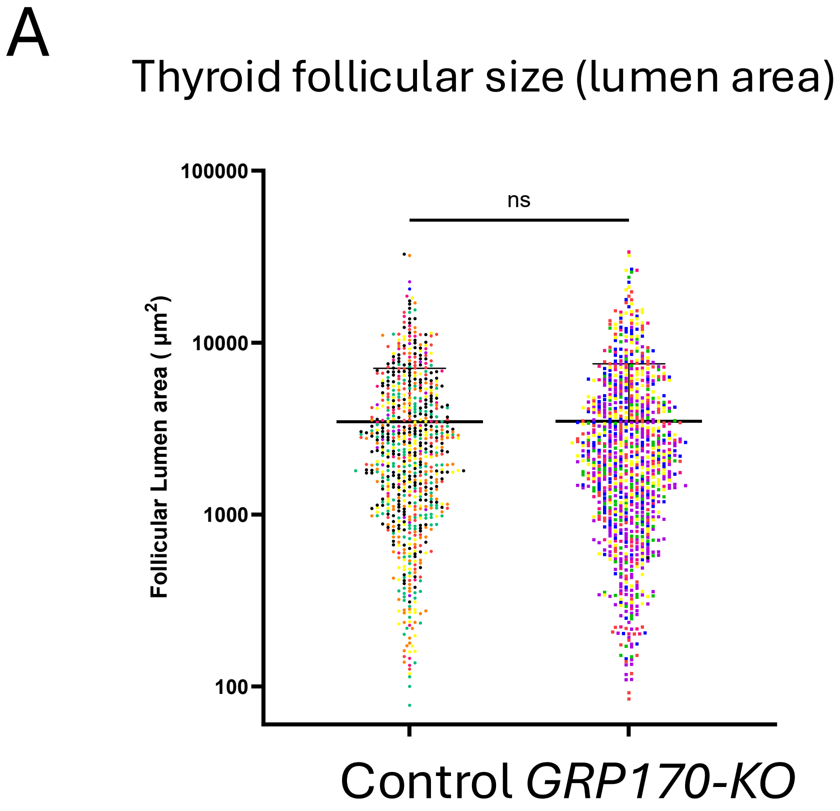

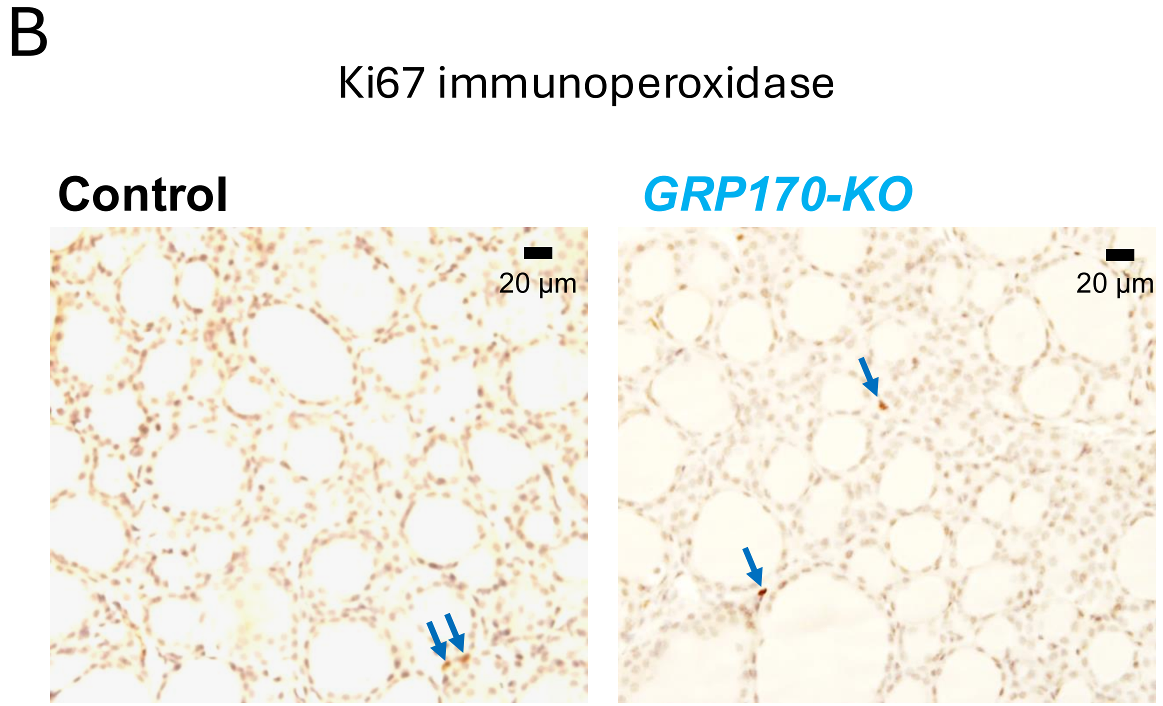

Zhang et al., Supplemental Figure S1

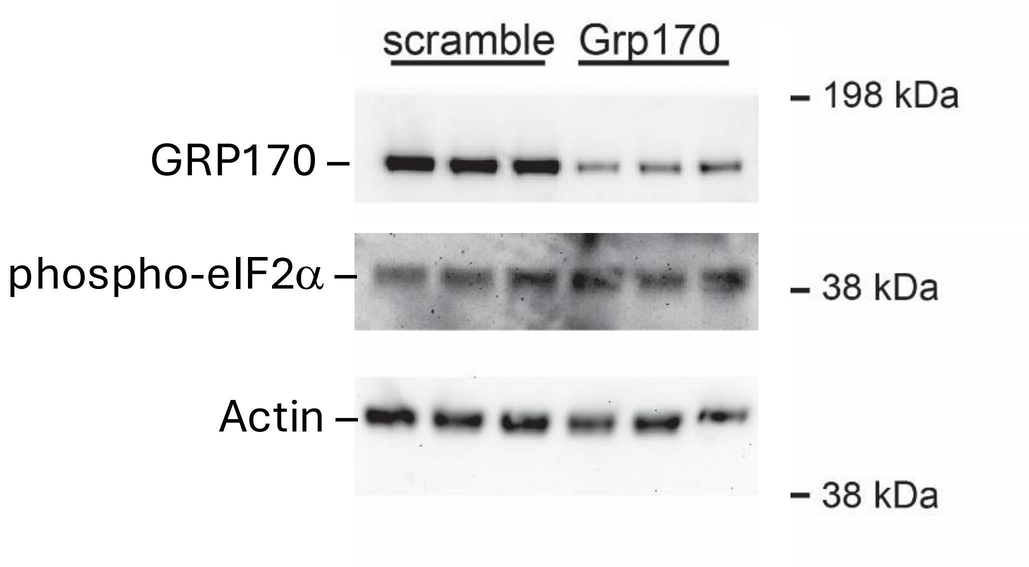

Note: these come from the same samples (and thus same actin blot) as Fig. 6A

Zhang et al., Supplemental Figure S2

C57BL6/j mice treated +/– PTU for 2 weeks

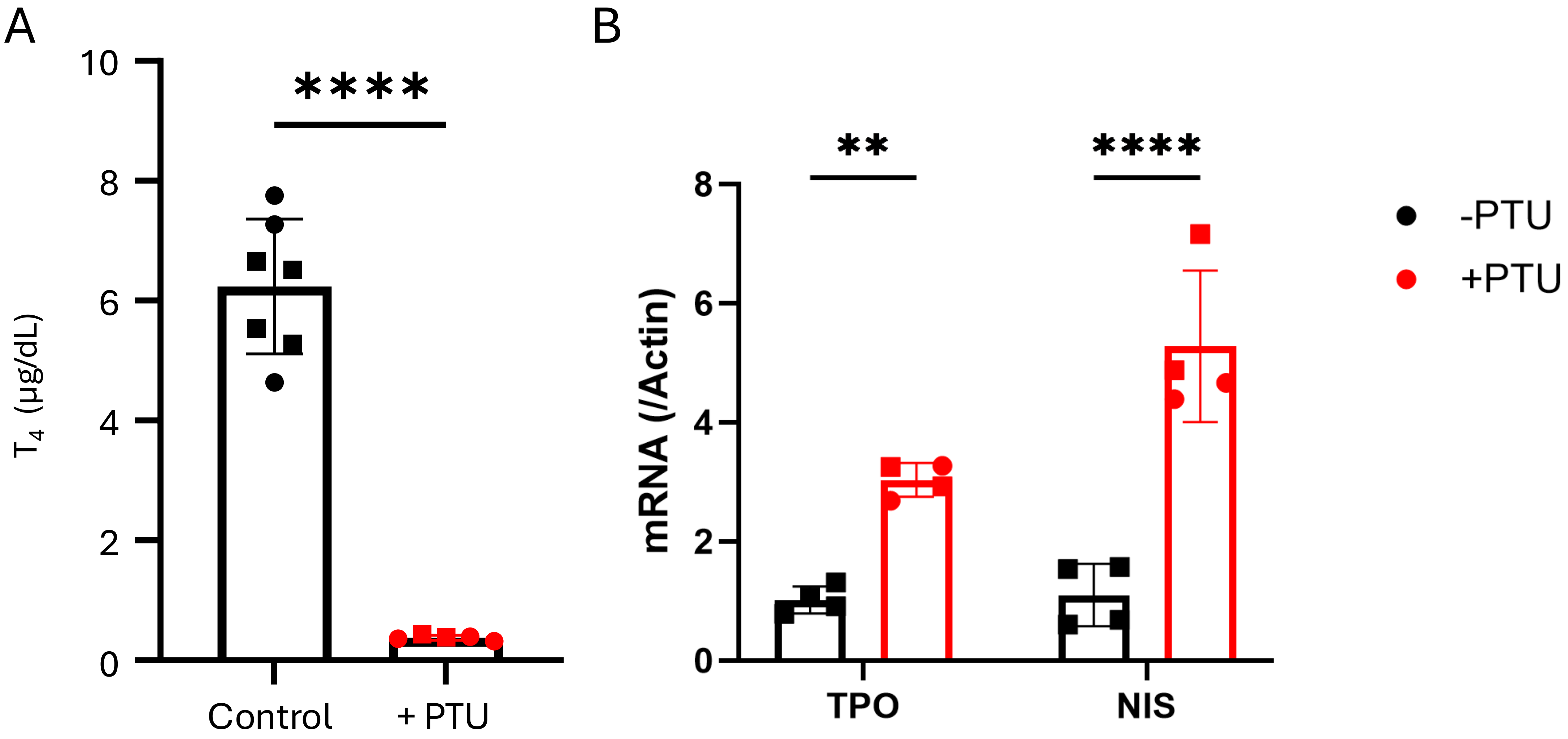

Zhang et al., Supplemental Figure S3

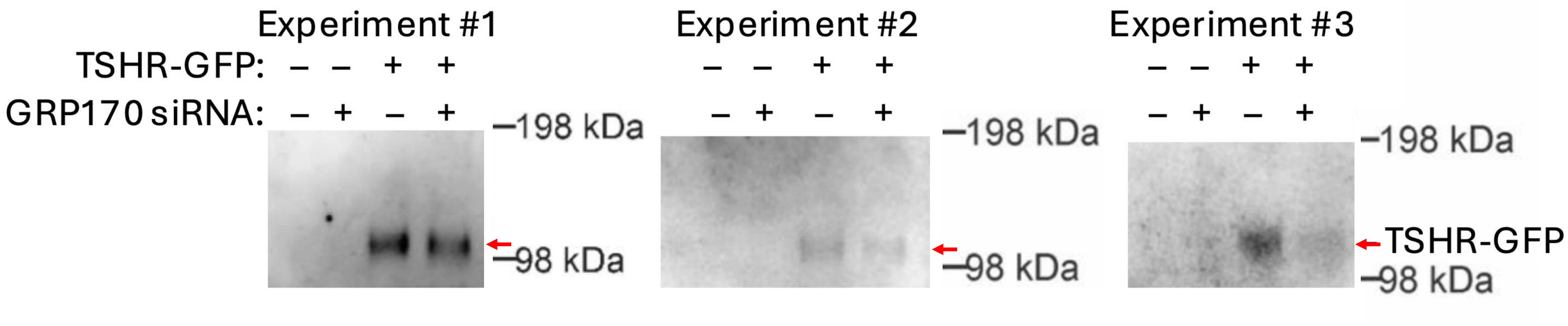

Zhang et al., Supplemental Figure S4
