# Supplementary material for: Thyroidal expression of ER molecular chaperone GRP170 is required for efficient TSH-mediated thyroid hormone synthesis
Source: JCI Insight. 2025 Sep 9;10(17):e191837. doi: 10.1172/jci.insight.191837 (PMC12487683; doi:10.1172/jci.insight.191837)

Fig. 2B Control: lane 1-3; lane 7-10
GRP170-KO: lane 4-6; lane 11-13

Lane 1-6 were shown in Fig. 2B. All lanes were quantified in Fig. 2C.
N=6-7

Shown in figure

Lane: 1 2 3 4 5 6 7 8 9 10 11 12 13

Brightfield

GRP170

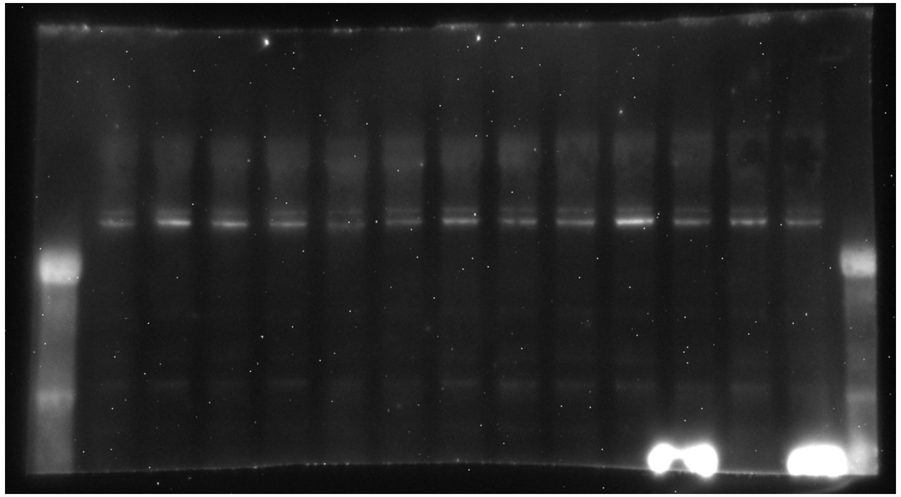

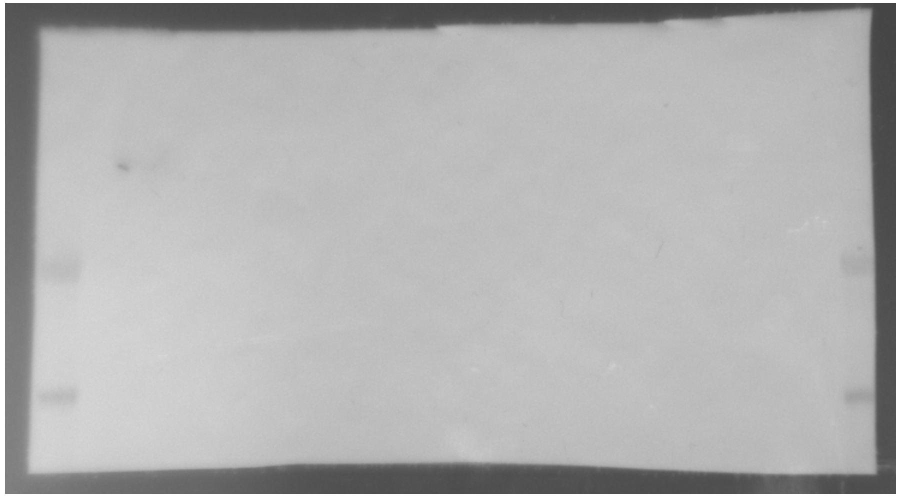

Lane: 1 2 3 4 5 6 7 8 9 10 11 12 13

ACTIN

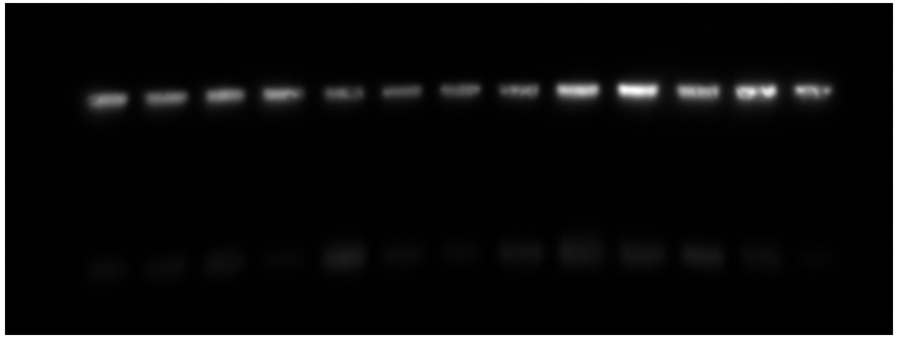

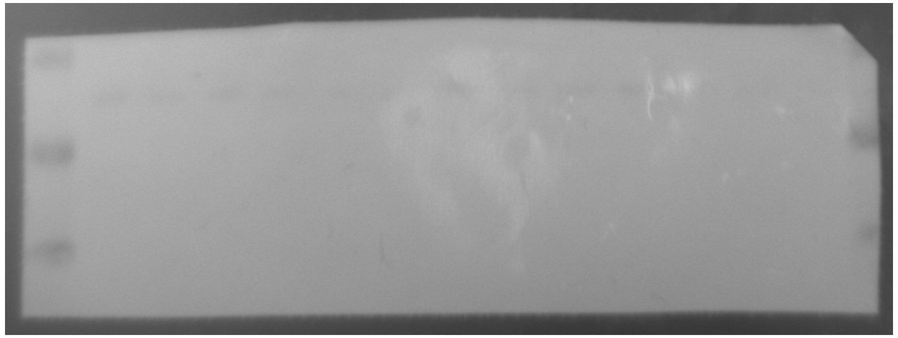

Fig. 2B, 3D and 4E were from the same gel.

Fig. 3D

Control: lane 1-4
GRP170-KO: lane 5-7

N=3-4

Shown in figure

Lane: 1 2 3 4 5 6 7

Brightfield

BiP

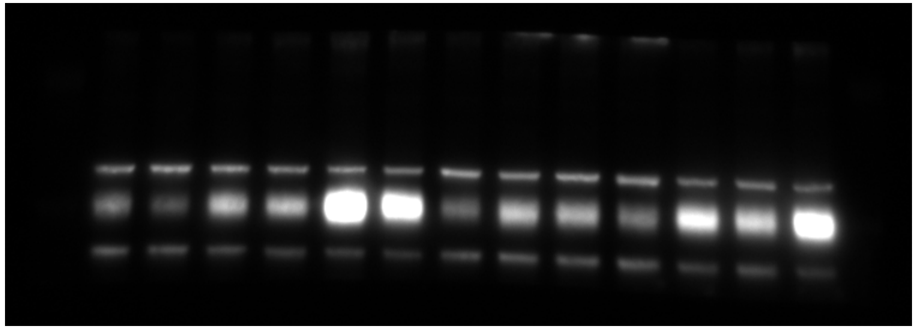

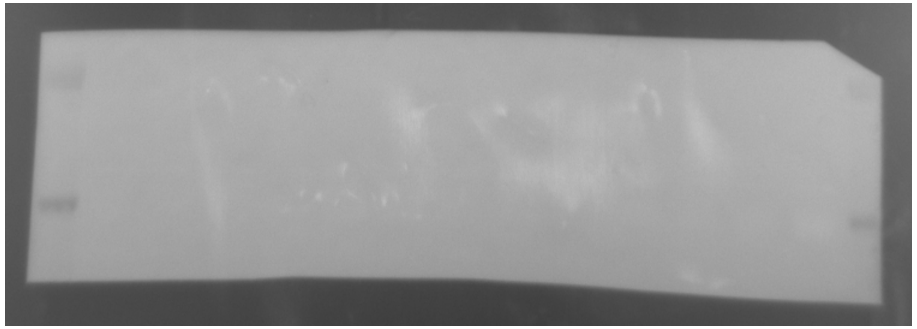

Lane: 1 2 3 4 5 6 7

ACTIN

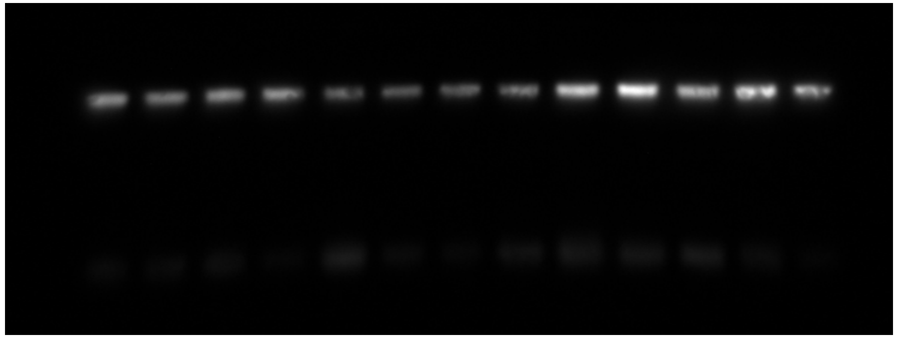

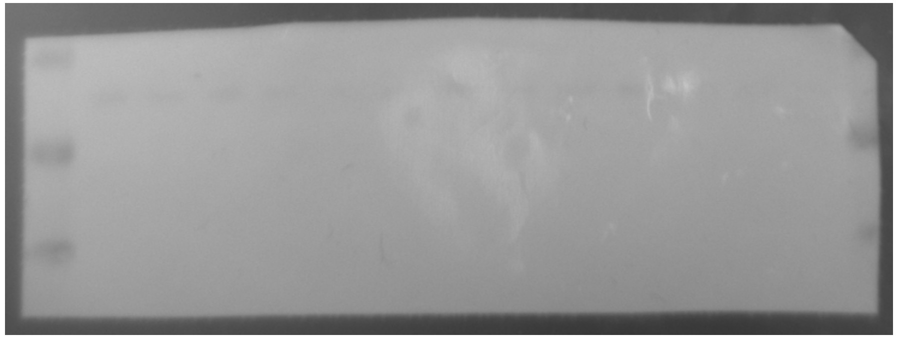

Fig. 2B, 3D and 4E were from the same gel.

Fig. 3E Control: lane 1, 2; lane 5, 6
GRP170-KO: lane 3, 4; lane 7, 8

Mock digestion: lane 1, 3, 5, 7
Endo H: lane 2, 4, 6, 8

Lane 5-8 were shown in Fig. 3E.
N=4

Shown in figure

Lane: 1 2 3 4 5 6 7 8

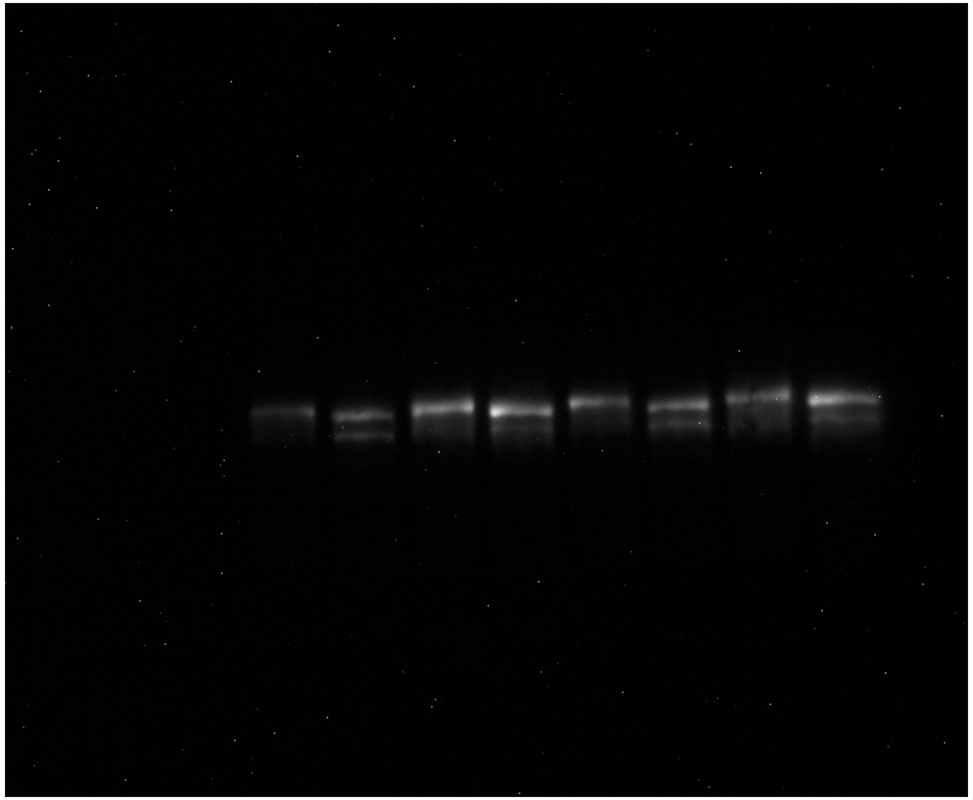

Brightfield

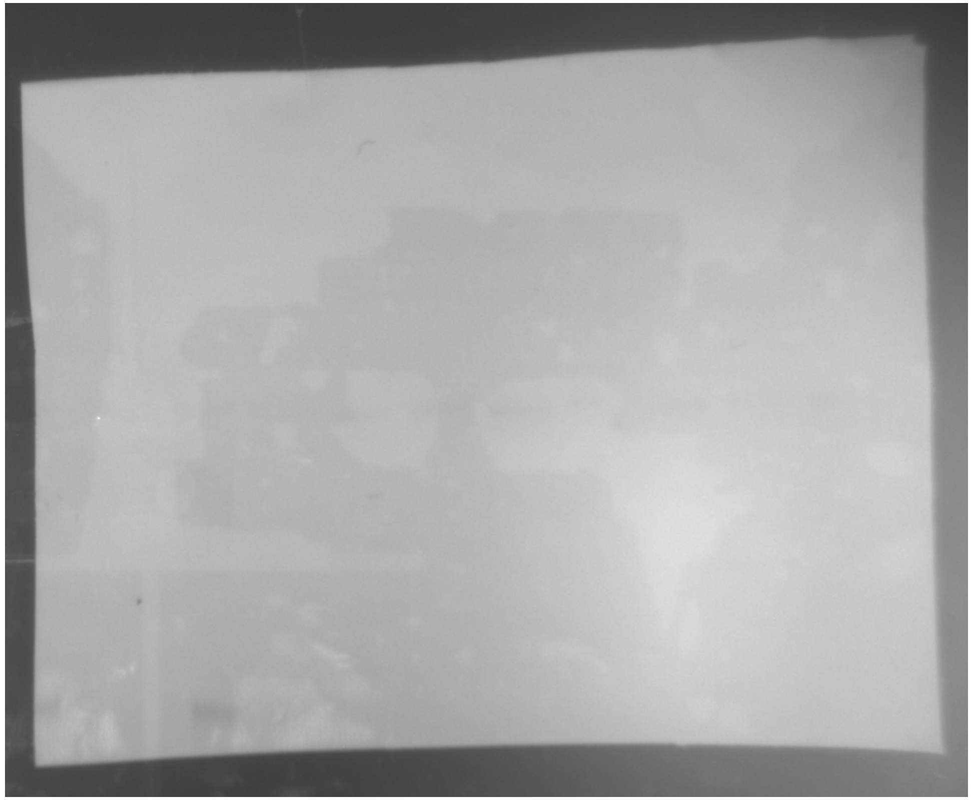

Fig. 4C

Control: lane 1-7
GRP170-KO: lane 8-13

N=6-7

Shown in figure

Brightfield

Lane: 1 2 3 4 5 6 7 8 9 10 11 12 13

TPO

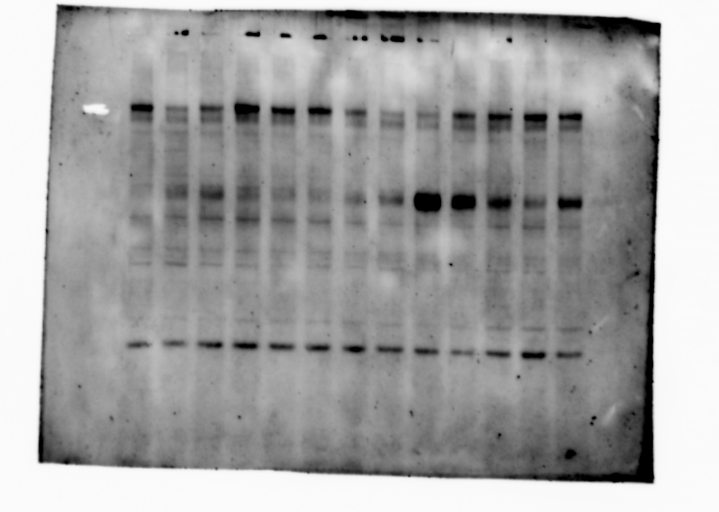

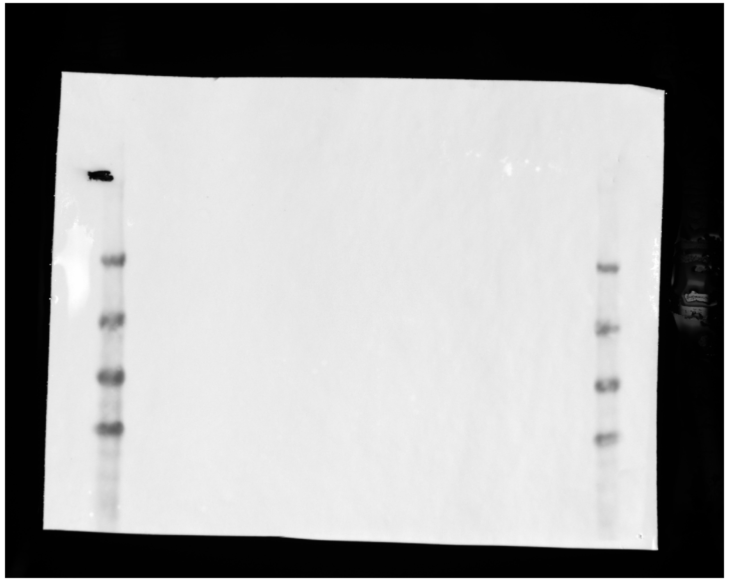

Lane: 1 2 3 4 5 6 7 8 9 10 11 12 13

ACTIN

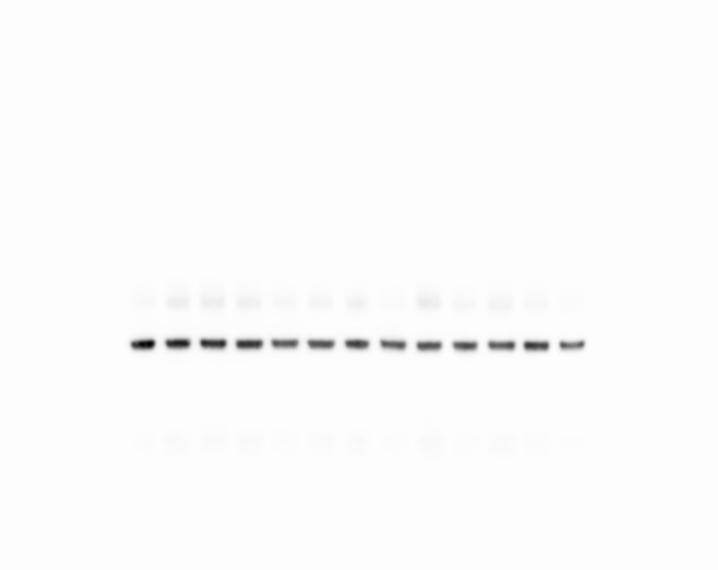

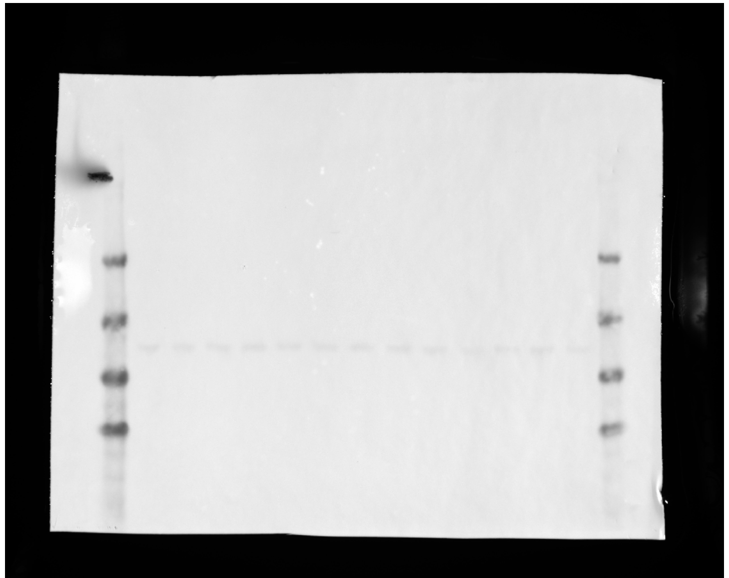

Fig. 4E Control: lane 1-3; lane 7-10

GRP170-KO: lane 4-6; lane 11-13

Lane 1-6 were shown in Fig. 4E. All lanes were quantified in Fig. 4F.

N=6-7

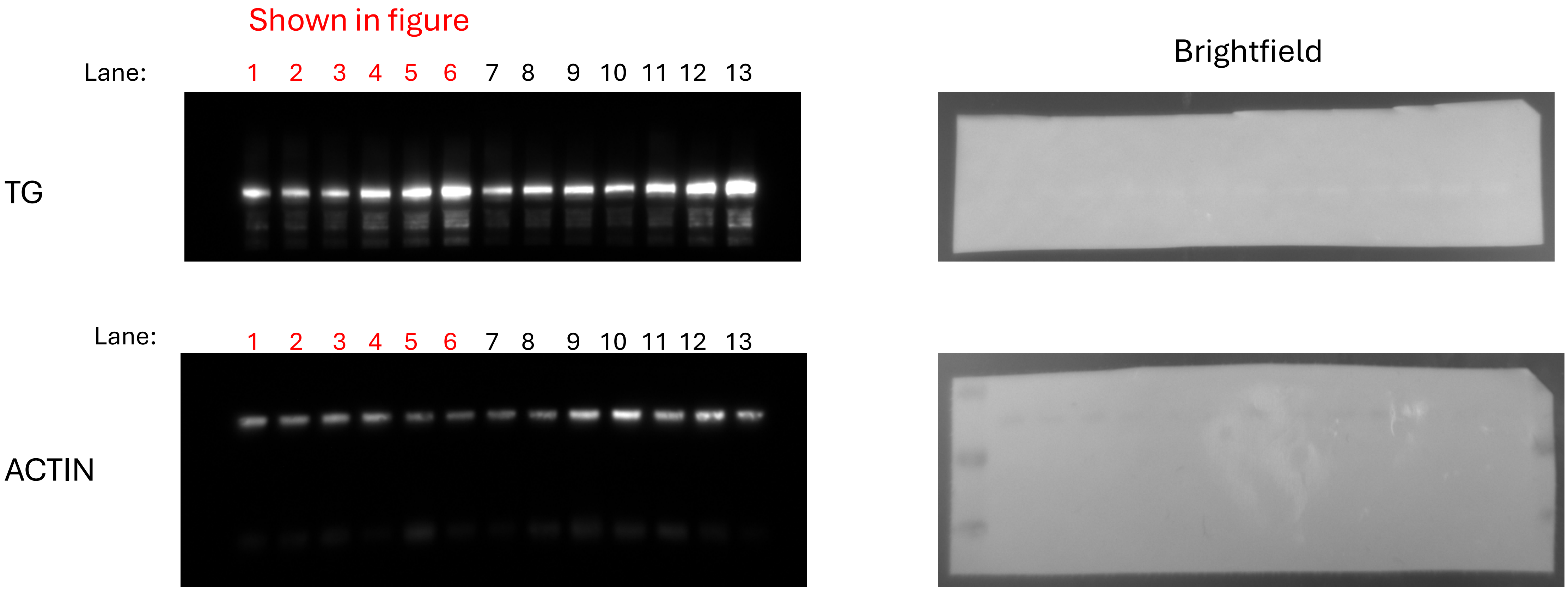

Fig. 2B, 3D and 4E were from the same gel.

Fig. 4G

Control: lane 1-4;
GRP170-KO: lane 5-7

N=4-6

Shown in figure

Lane: 1 2 3 4 5 6 7

T4

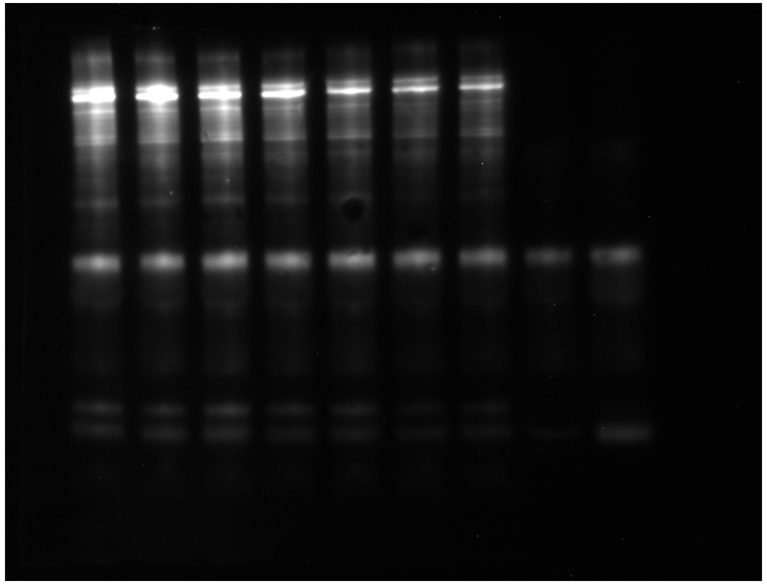

Brightfield

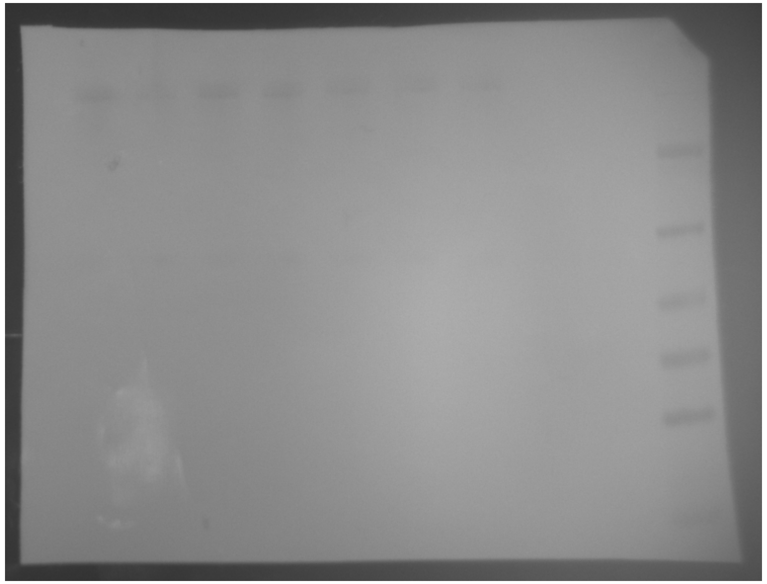

Lane: 1 2 3 4 5 6 7

Tg

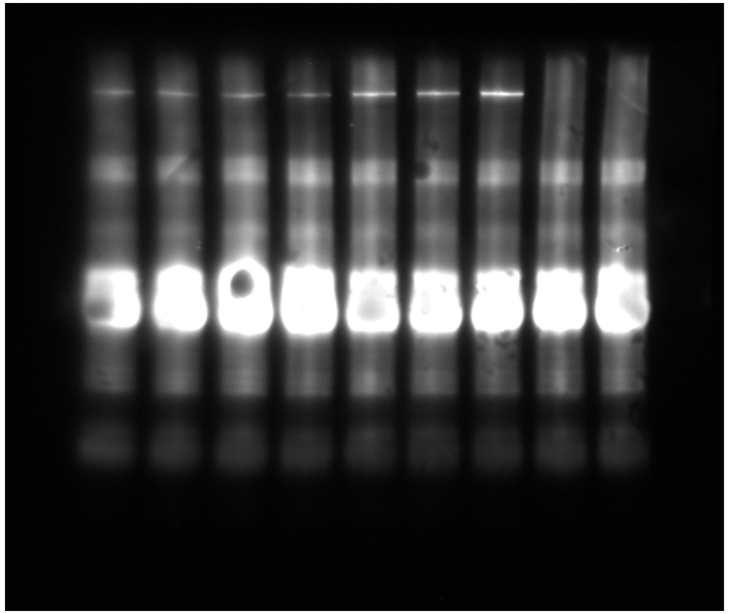

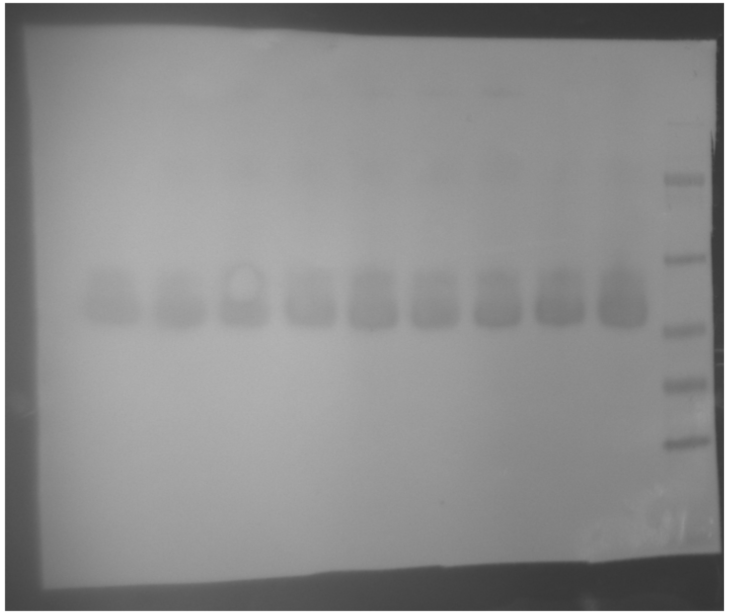

Fig. 5A

Control: lane 1-7
GRP170-KO: lane 8-13

N=6-7

Shown in figure

Lane: 1 2 3 4 5 6 7 8 9 10 11 12 13

NIS

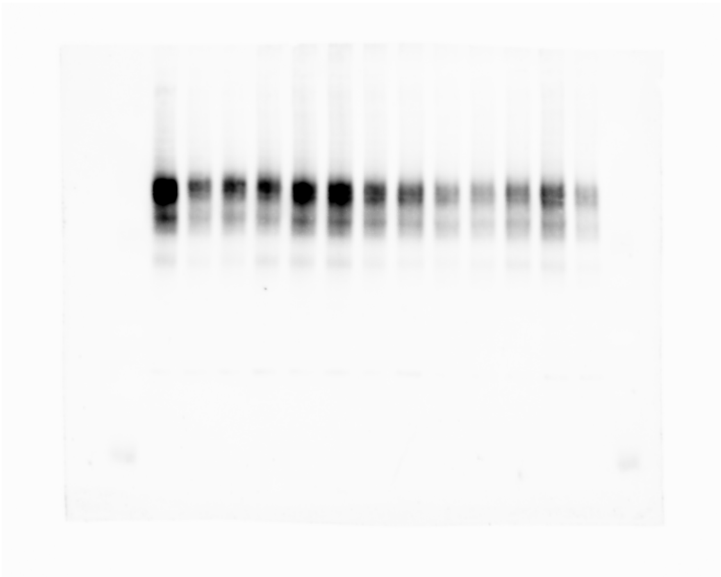

Brightfield

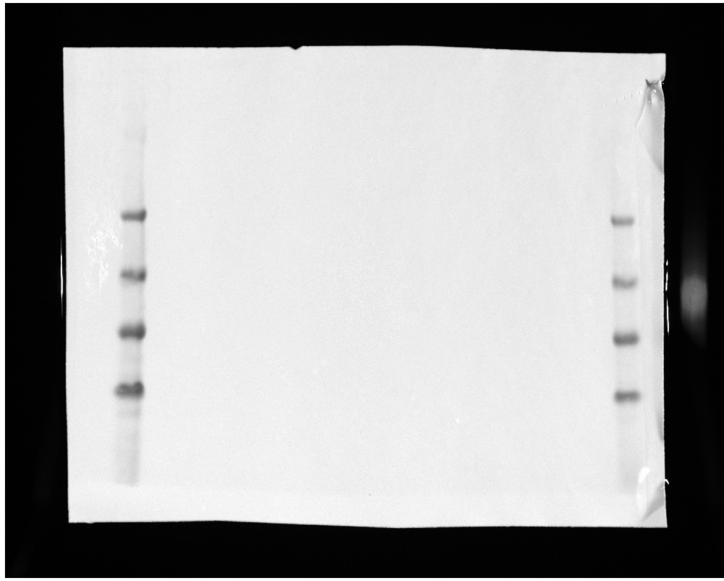

Lane: 1 2 3 4 5 6 7 8 9 10 11 12 13

ACTIN

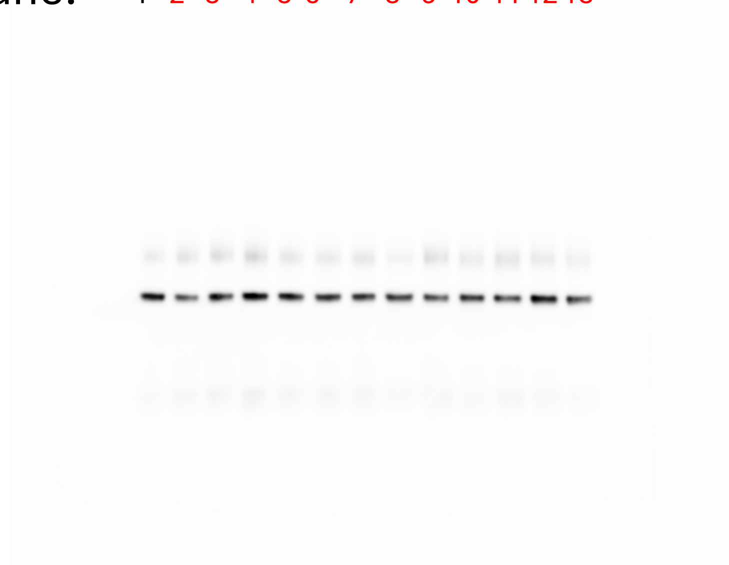

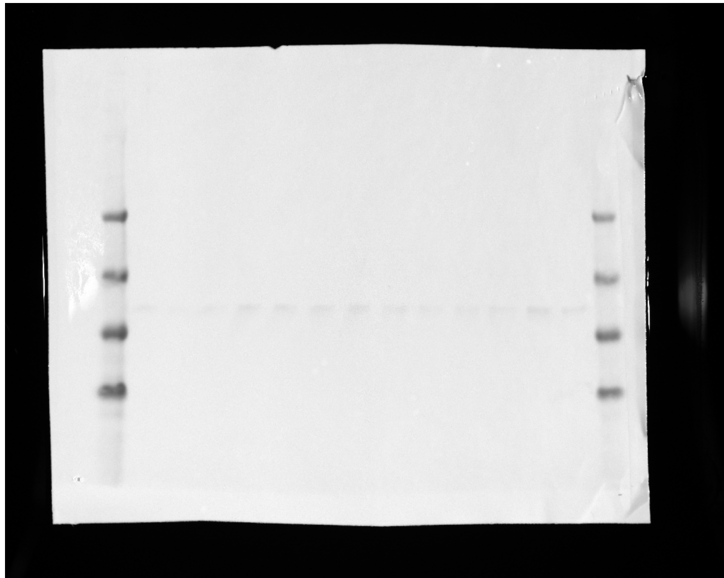

Fig. 5D

Control: lane 1-7;
GRP170-KO: lane 8-13

N=5-7

Shown in figure

Lane: 1 2 3 4 5 6 7 8 9 10 11 12 13

TSHR

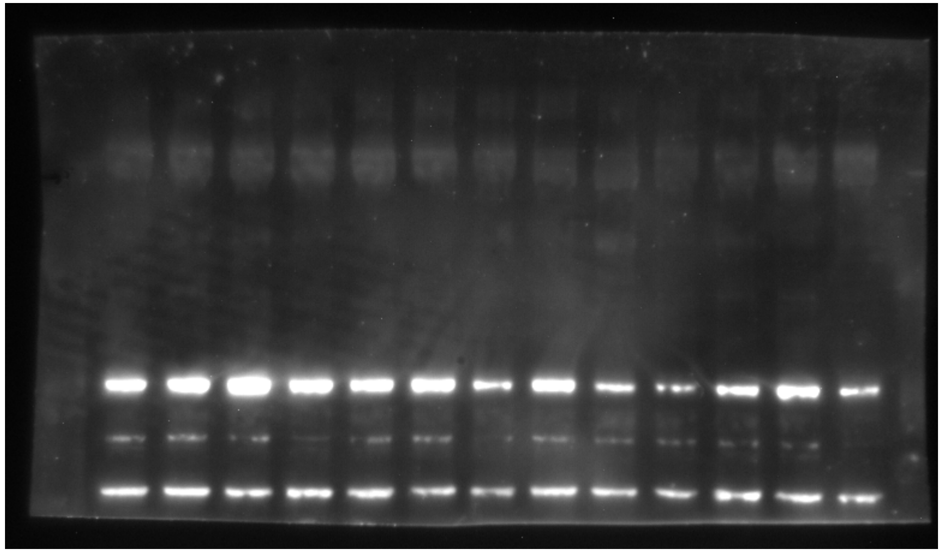

Brightfield

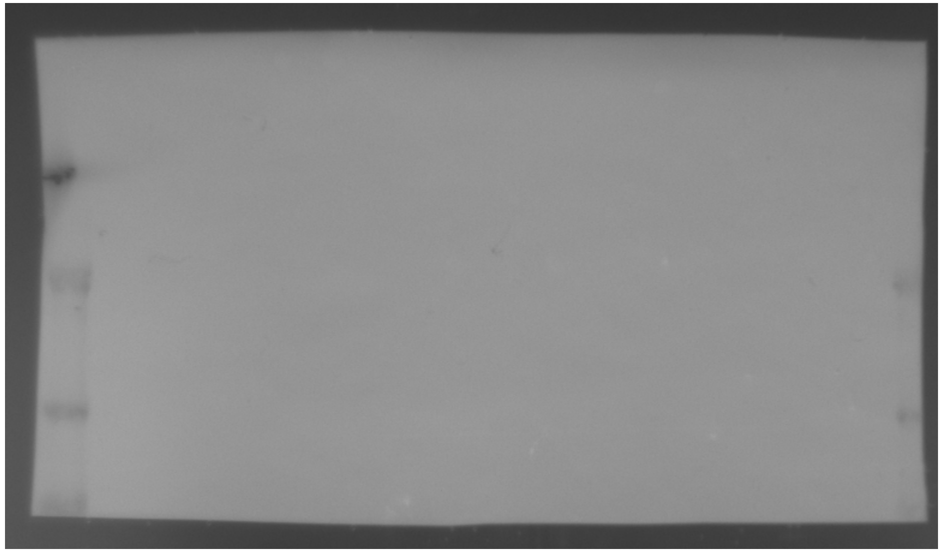

Lane: 1 2 3 4 5 6 7 8 9 10 11 12 13

ACTIN

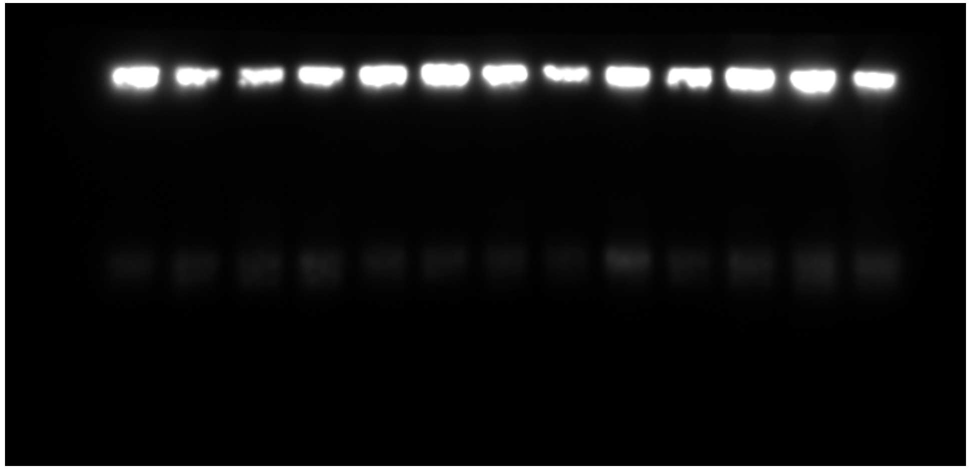

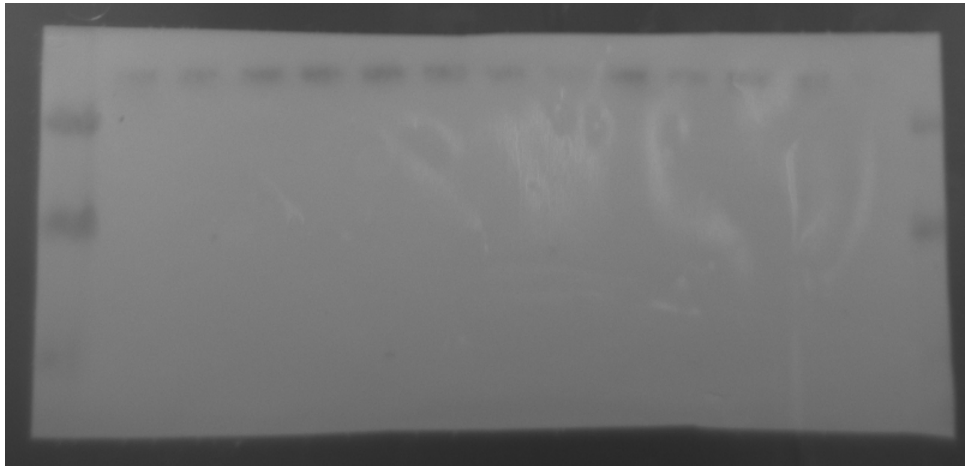

Lane 8 was an outlier.

Fig. 6A

scramble: lane 1-3; lane 7-9
GRP170-KD: lane 4-6; lane 10-12

N=3

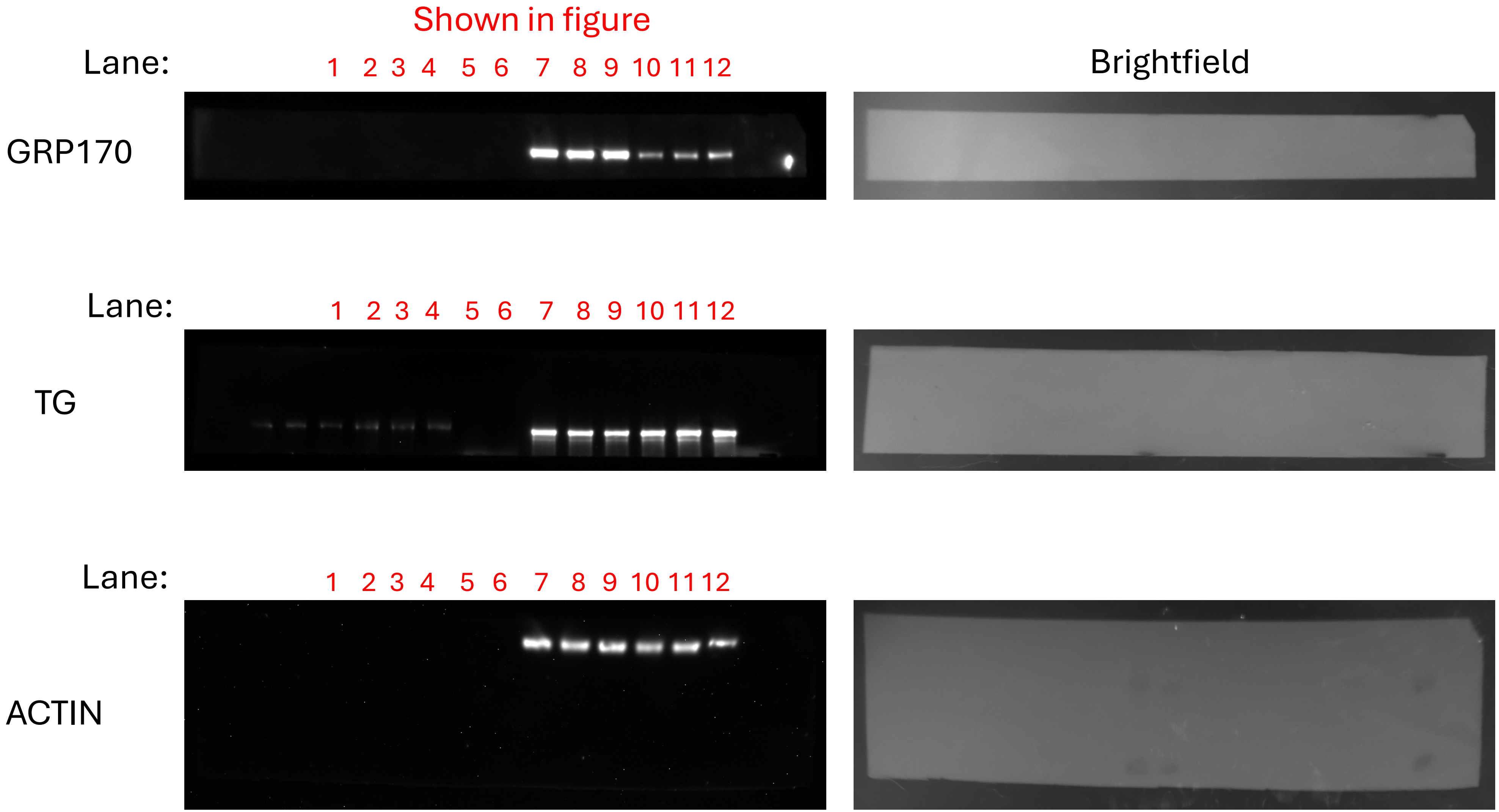

Fig. 6C

scramble: lane 1-3
GRP170-KD: lane 4-6

N=3

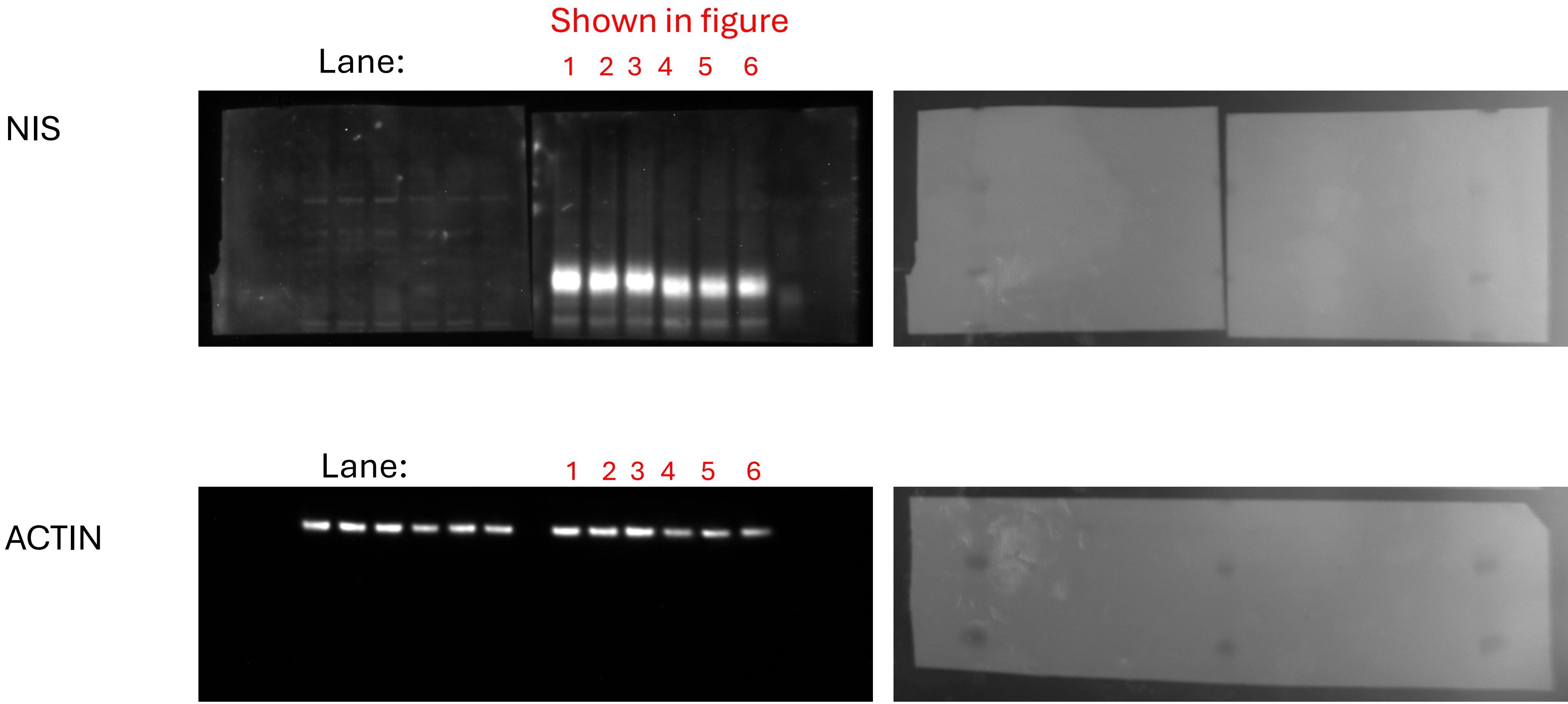

Fig. 6D

scramble: lane 1-3; lane 7-9
GRP170-KD: lane 4-6; lane 10-12

Lane 1-6 were shown in Fig. 5D.

n=4

Mock: lane 1, 4, 7, 10
Endo H: lane 2, 5, 8, 11
PNGase F: lane 3, 6, 9, 12

Shown in figure

Lane: 1 2 3 4 5 6 7 8 9 10 11 12

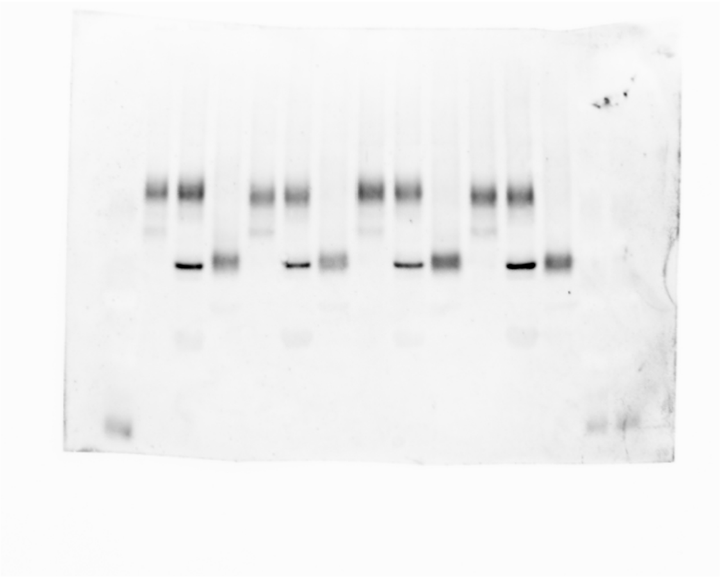

Brightfield

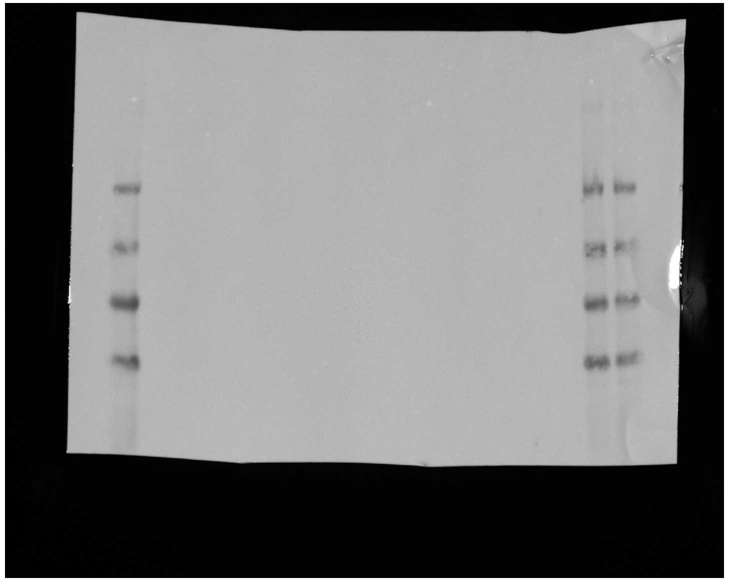

Fig. 6E N=4

Experiment 1

scramble: lane 1-2; lane 6-8
GRP170-KD: lane 3-5; lane 9-11

-TSHR: lane 1-5
+TSHR: lane 6-11

Mock: lane 1, 3, 6, 9
Endo H: lane 2, 4, 7, 10
PNGase F: lane 5, 8, 11

Shown in figure 3-8 % gel

Brightfield

Lane: 1 2 3 4 5 6 7 8 9 10 11

GFP

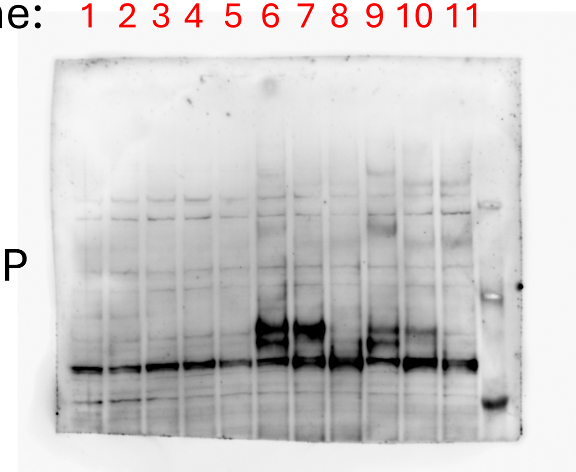

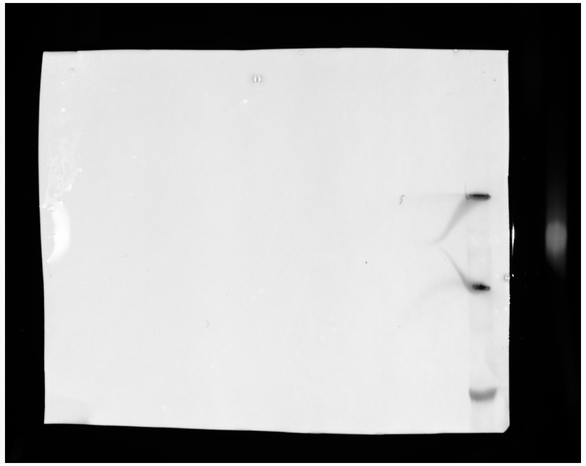

4-12 % gel

Lane: 1 2 3 4 5 6 7 8 9 10 11

GFP

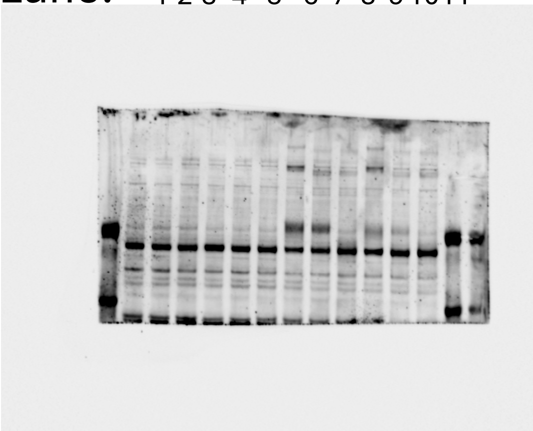

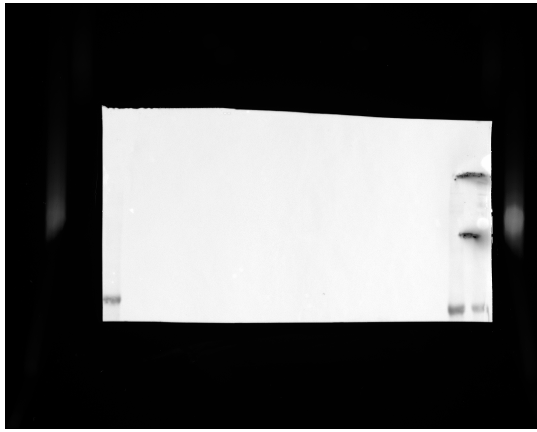

Lane: 1 2 3 4 5 6 7 8 9 10 11

ACTIN

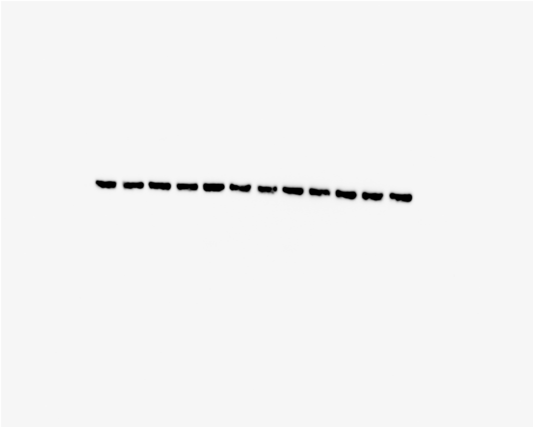

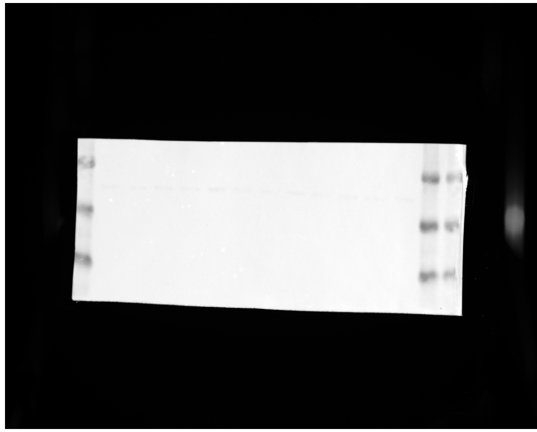

Brightfield

Fig. S2

scramble: lane 1-3
GRP170-KD: lane 4-6

N=3

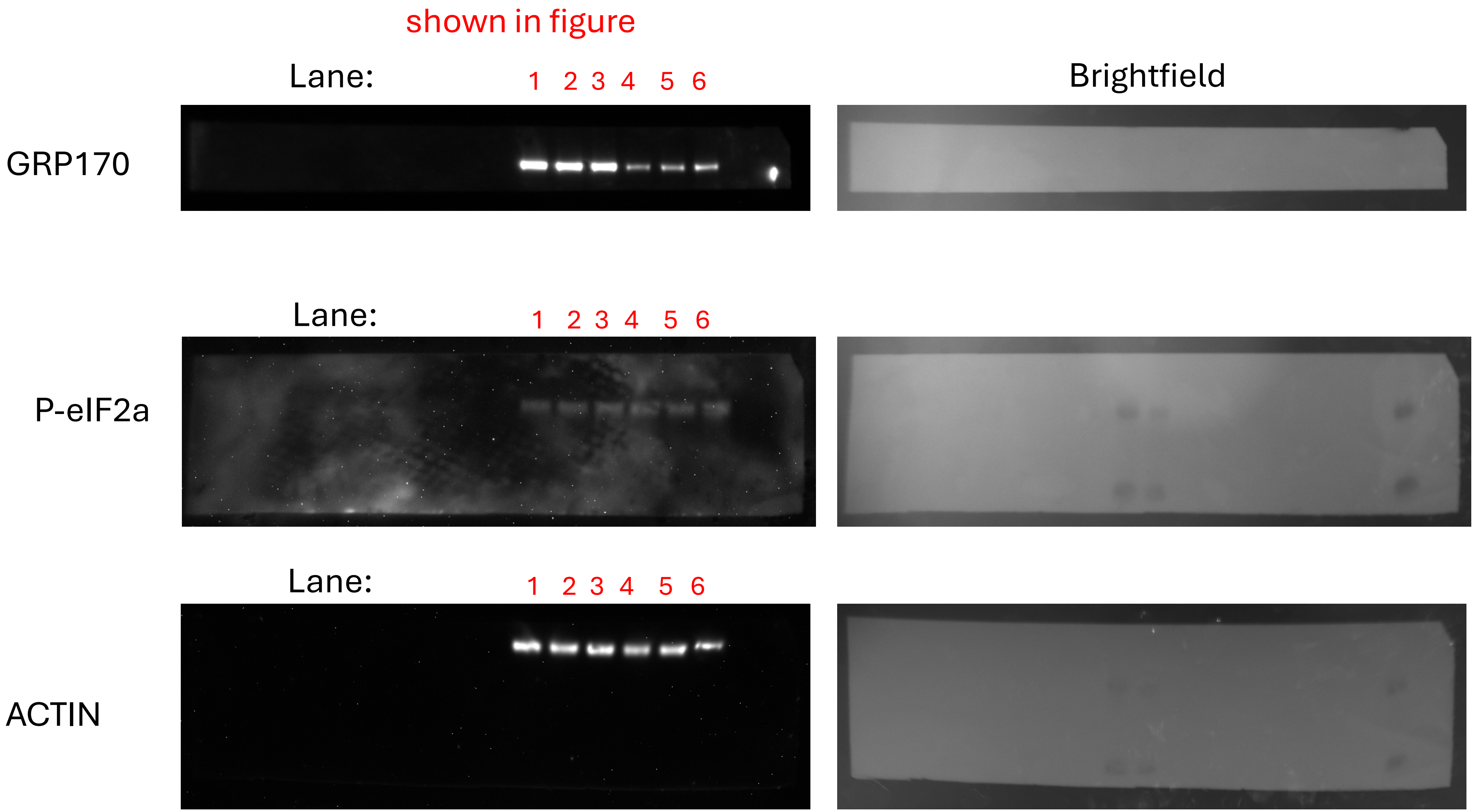

these come from the same samples (and thus same Grp170 and actin blot) as Fig. 6A

Fig. S4 N=3

Lane 1: -TSHR, scramble
Lane 2: -TSHR, GRP170-KD
Lane 3: +TSHR, scramble
Lane 4: +TSHR, GRP170-KD

Experiment 1

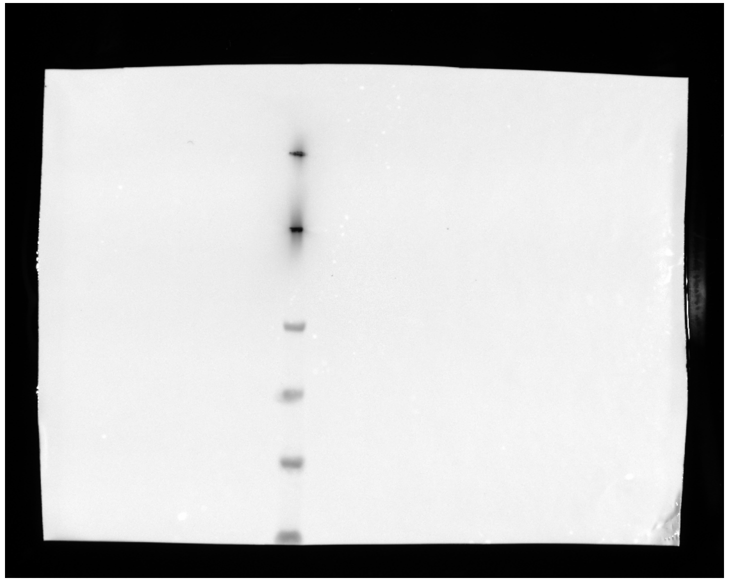

Membranes
on this page;
blots on final page

Experiment 2

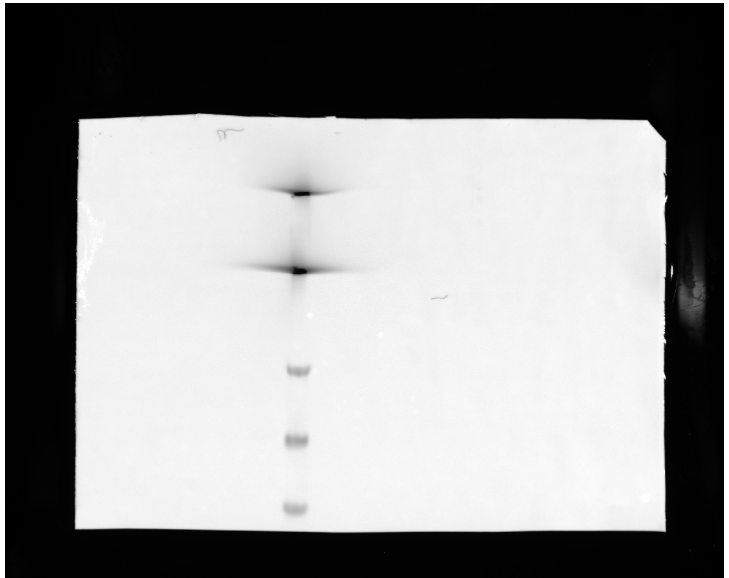

Fig. S4

Lane 1: -TSHR, scramble
Lane 2: -TSHR, GRP170-KD
Lane 3: +TSHR, scramble
Lane 4: +TSHR, GRP170-KD

Experiment 3

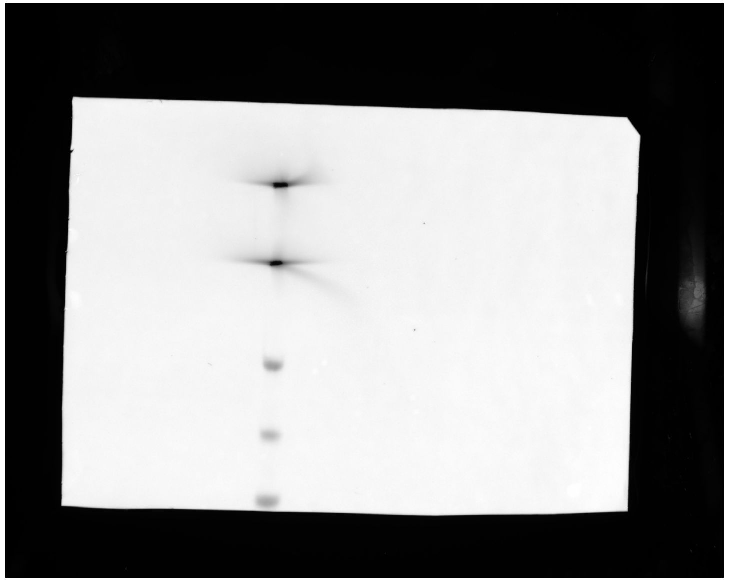

Membranes
on this page;
blots on final page

Sample:
1. negative control - no lysate
2. no TSHR, scamble
3. no TSHR, Grp170-KD
4. +TSHR, scamble
5. +TSHR, Grp170-KD

anti-GFP
(rabbit icllabs)

Transfection + biotinylation #1

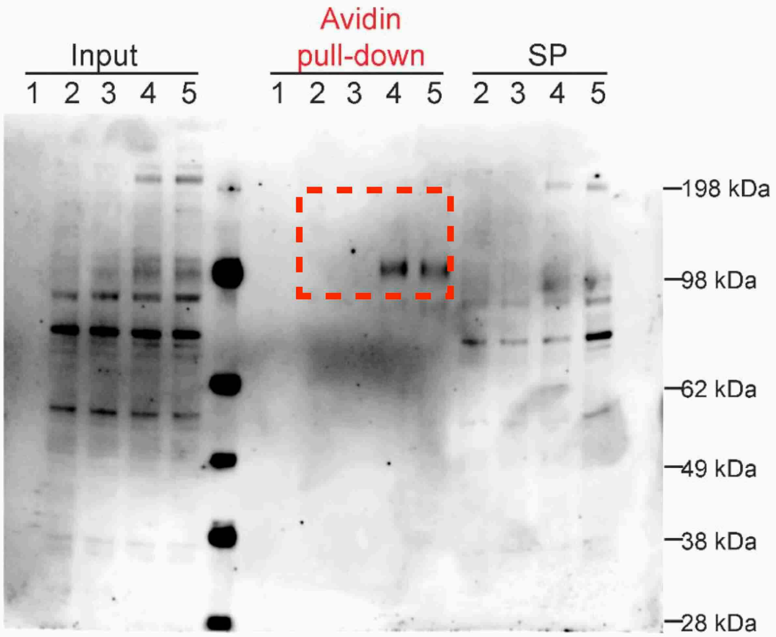

Fig. S4

Lane:
1. negative control - no lysate
2. no TSHR, scamble
3. no TSHR, Grp170-KD
4. +TSHR, scamble
5. +TSHR, Grp170-KD

anti-GFP
(rabbit icllabs)

Transfection + biotinylation #2

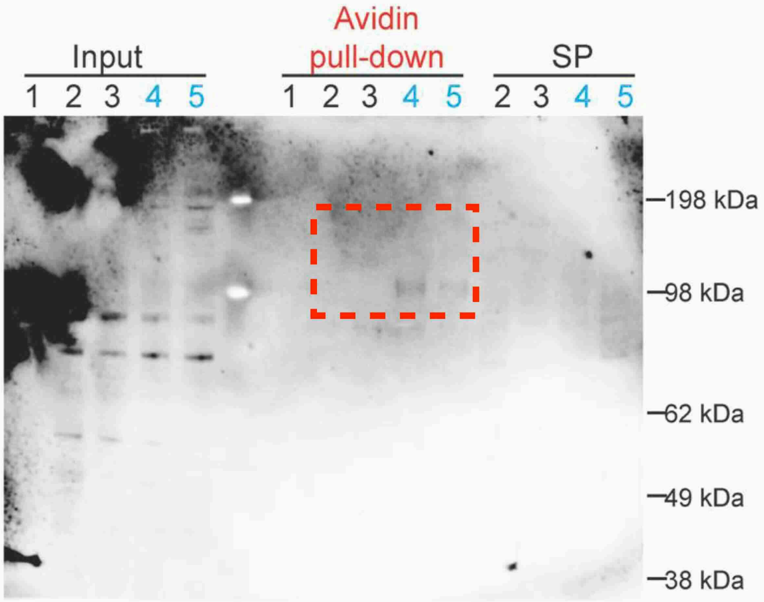

Lane:
1. negative control - no lysate
2. no TSHR, scamble
3. no TSHR, Grp170-KD
4. +TSHR, scamble
5. +TSHR, Grp170-KD

anti-GFP
(rabbit icllabs)

Transfection + biotinylation #3

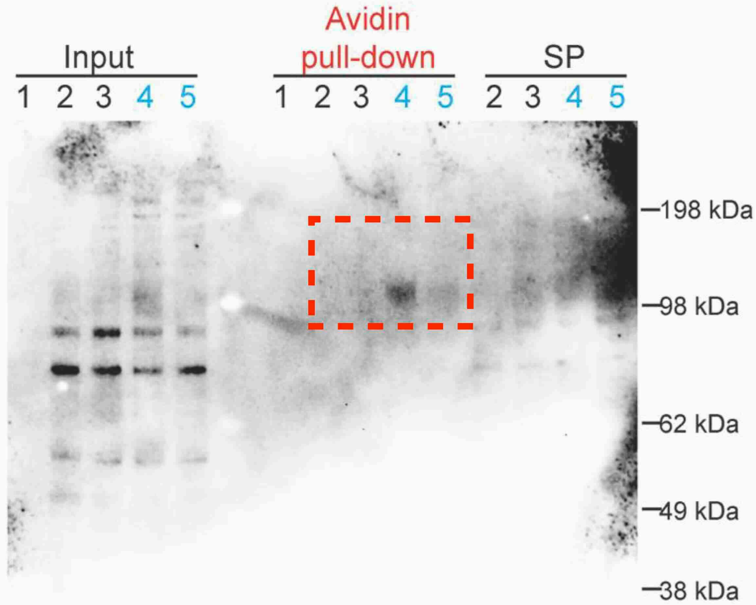

Supplement: Unedited blot and gel images [file jciinsight-10-191837-s109.pdf]
